# Reconciling contradictory models of subthalamic nucleus contributions to basal ganglia beta oscillations

**DOI:** 10.1371/journal.pcbi.1013942

**Published:** 2026-08-17

**Authors:** Ka Nap Tse, G. Bard Ermentrout, Jonathan E. Rubin

**Affiliations:** Department of Mathematics, University of Pittsburgh, Pittsburgh, Pennsylvania, United States of America; The University of Iowa College of Engineering, UNITED STATES OF AMERICA

## Abstract

Recent computational studies of Parkinson’s disease have yielded contradictory findings regarding the role of the subthalamic nucleus (STN) in pathological beta oscillations, with some models implicating STN as essential for beta generation and others suggesting that STN suppresses oscillations. This work addresses these discrepancies by systematically investigating how the specific features of the integrate-and-fire neurons used in these models influence simulated basal ganglia network dynamics. Using both rate models and spiking network simulations incorporating coupled subthalamopallidal and pallidostriatal circuits, we demonstrate that the choice between leaky integrate-and-fire (LIF) and quadratic integrate-and-fire (QIF) models to represent STN neurons fundamentally impacts the phase relationship between STN and external globus pallidus prototypical (Proto) neuron populations. QIF STN neurons establish in-phase coupling with Proto neurons, which enhances beta oscillation amplitude, while LIF STN neurons develop anti-phase relationships that suppress beta power. Through intervention experiments and parameter sweeps across physiologically relevant firing rates, we show that these phase-related effects persist robustly across network conditions, and we mathematically establish conditions under which these results are guaranteed to hold. Our findings reveal that the fundamental mathematical structure underlying spike generation, rather than other biophysical details, determines whether the subthalamopallidal loop acts as a beta amplifier or suppressor. This mechanistic insight reconciles contradictory findings in the literature, demonstrates that seemingly minor modeling choices can have profound consequences for understanding disease mechanisms and therapeutic targets, and offers predictions for determining which model framework reflects the biological reality.

## Author summary

Substantial work has explored the mechanisms underlying enhanced beta oscillations in the basal ganglia, motivated by their potential relevance to parkinsonian conditions and associated treatments. Often inferences about these mechanisms are based on simulations and reasoning that focus on features of network connectivity. We show that in fact the specific dynamical properties of the neurons in these circuits can strongly influence their emergent dynamics, with completely opposing effects arising in a given network structure depending on which neuron model is used, and we explain the factors underlying this divergence. Based on these factors, the determination of a small set of neuron properties in future biological experiments will lead to predictions about the mechanisms that can generate beta oscillations in the parkinsonian basal ganglia.

## Introduction

The basal ganglia network comprises interconnected subcortical nuclei including the striatum with its spiny projection neurons and fast-spiking interneurons [1–3], the subthalamic nucleus (STN), the external globus pallidus (GPe) with its prototypical and arkypallidal neuron populations [4,5], and the substantia nigra. These circuits interact through what are often called the direct and indirect pathways, resulting in dynamic neural outputs that contribute to flexible motor control [6,7]. Parkinson’s disease (PD) is characterized by the loss of dopaminergic neurons in the substantia nigra, leading to motor symptoms including tremor, rigidity, and bradykinesia [8]. A hallmark of the disease is the emergence of abnormal beta oscillations (13–30 Hz) within basal ganglia circuits [9], which correlate with motor symptom severity and are suppressed by effective deep brain stimulation [10–13]. Recent insights reveal that pathological beta manifests as intermittent “beta bursts” that correlate more strongly with symptoms than do average beta power [14,15].

Two competing hypotheses have emerged regarding mechanisms of beta generation in the basal ganglia. The first proposes that STN-GPe interactions form a critical pacemaker unit [16], supported by evidence that subthalamic activity correlates strongly with downstream basal ganglia activity in parkinsonian conditions [17,18]. This excitatory-inhibitory network architecture is well-established to support oscillatory dynamics [19]. Foundational computational models demonstrate how subthalamopallidal networks may generate parkinsonian oscillations [20–25], with frequency related to slow potassium-dependent adaptation currents in the STN [26]. However, recent evidence has challenged the traditional STN-centric view, with opto-inhibition studies showing that a peak of beta power remains in the GPe LFP even after STN suppression [27]. As an alternative, experimental evidence from rodent studies suggests that pallidostriatal pathways may be the primary driver of beta oscillations [27–34], with computational models showing that these connections promote beta oscillations in dopamine-depleted networks [35]. Other studies have also implicated sites outside of the STN and GPe, namely cortical [36] and striatal [37,38] circuits, in beta generation.

Most puzzling are contradictory results from recent modeling work. One computational study [39] demonstrated that dopamine depletion leads to pathological beta synchronization with STN activity playing an essential role for beta generation. Another from the same time frame [40] found that pallidostriatal circuit changes can trigger beta synchrony with STN actually weakening rather than promoting oscillations. This contradiction is particularly perplexing given that both studies employed similar circuit architectures and addressed the same fundamental question.

This work addresses these contradictory findings by systematically investigating how integrate-and-fire model selection influences simulated basal ganglia network dynamics. We demonstrate that differences in neuronal model implementation can fundamentally alter the predicted role of STN in beta generation, potentially explaining the conflicting results in recent literature. Our findings suggest that the mathematical structure underlying spike generation, rather than specific biophysical details, determines phase relationships and oscillation amplitudes within basal ganglia circuits.

The remainder of this paper is organized as follows. Section 2 introduces a simplified rate model to demonstrate how the phase relationship between STN and GPe prototypical neuron populations directly influences beta oscillation amplitude. Section 3 analyzes single neuron dynamics under periodic inhibition, using *time-to-spike functions* to prove that LIF and QIF neurons exhibit fundamentally different phase preferences: anti-phase and in-phase, respectively. Section 4 presents comprehensive spiking network simulations incorporating both pallidostriatal and subthalamopallidal circuits, demonstrating that the single-neuron phase preferences scale to population-level dynamics and determine whether STN enhances or suppresses beta oscillations. Section 5 discusses implications of our findings and directions for future research. Mathematical proofs supporting the analytical results are provided in the Supporting Information, Appendices A and B in S1 Appendix.

## Models and methods

All code for the models and simulations presented in this paper along with associated documentation is openly available in a GitHub repository at the following site: https://github.com/kanaptse/BG-Oscillation-Synergy-Suppression.

### Population firing rate model

We use a population firing rate model to provide a simplified setting in which to demonstrate the effect of phase relationships on beta oscillations. Let *P*={STN, Proto, FSI, D2} be the set containing all populations in the model, which are subthalamic nucleus neurons, prototypical neurons of the external segment of the globus pallidus or GPe, striatal fast spiking interneurons, and striatal spiny projection neurons with D2 dopamine receptors (the predominant source of striatal inputs to the GPe), respectively. The rate dynamics for population p∈P obeys the equation


$$\begin{array}{c} \tau_{p}\frac{dr_{p}}{dt}=-r_{p}+a_{p}f\left(b_{p}\left(\sum_{q\in P}g_{p\leftarrow q}r_{q}(t-d_{p\leftarrow q})+I_{p}\right)+c_{p}\right), \end{array}$$ (1)

where *f* is the sigmoid function *f*(*x*)=1/(1 + *e*^-*x*^). The population-specific parameters that we use in equation (1) are provided in Table 1, while connection strengths are detailed in Table 2. Connection strengths not explicitly listed in the table are set to zero. Note in particular that there is no reciprocal excitation between STN neurons, consistent with current experimental findings. All parameters have been calibrated to match the physiological firing rates of each population: STN, 12–20 Hz [41]; Proto, 40–60 Hz [5]; FSI, 10–20 Hz [42, 43]; and D2, 0.5–2.5 Hz [44]. Specifically, $c_{p}$ sets a firing rate in the absence of inputs (i.e., with $b_{p}=0$), $b_{p}$ sets a sensitivity to inputs, $a_{p}$ is a gain scaling parameter, and $I_{p}$ serves as an extra tuning parameter. Among all possible transmission delays, we specifically vary only $d_{\text{STN}\leftarrow \text{Proto}}$ to control the phase relationship between STN and Proto populations, the impact of which is the primary focus of this preliminary part of our study. All other delays are set to zero. It is important to note that this manipulation does not represent any physiological mechanism; rather, it serves as a computational tool to systematically explore how different phase relationships affect network dynamics. In reality, phase relationships in the basal ganglia are determined by many factors. In the Results section, we will examine how intrinsic neuronal dynamics can modulate these phase relationships.

**Table 1:** Parameter values for the firing rate model for the basal ganglia populations considered. The parameters $a_{p}$, $b_{p}$, $c_{p}$, $I_{p}$, and $\tau_{p}$ are shown for each neural population (STN, Proto, FSI, and D2), along with the target average firing rate ranges that these parameters were calibrated to achieve.

Population	$a_{p}$	$b_{p}$	$c_{p}$	$I_{p}$	$\tau_{p}$	Target avg. rate (Hz)
STN	31.3	0.127	2.33	0	12	12–20
Proto	108	0.0198	2.33	-30	10	40–60
FSI	41.6	0.0327	5.28	-20	20	10–20
D2	6.61	0.0326	5.28	-80	20	0.5–2.5

**Table 2:** Connection strengths between populations in the basal ganglia model. Each $g_{p\leftarrow q}$ represents the strength of the connection from population *q* to population *p*. These parameter values were selected to achieve the target firing rates specified in Table 1.

Connection	Value
$g_{\text{STN}\leftarrow \text{Proto}}$	-0.4
$g_{\text{Proto}\leftarrow \text{D2}}$	-50
$g_{\text{Proto}\leftarrow \text{STN}}$	4
$g_{\text{FSI}\leftarrow \text{Proto}}$	-4
$g_{\text{D2}\leftarrow \text{FSI}}$	-10

### Phase difference analysis

Phase differences between Proto and STN populations in this paper were calculated using Fast Fourier Transform (numpy.fft.fft) analysis. The firing rate signals were demeaned and transformed to the frequency domain. The dominant frequency was identified as the frequency with maximum power in the Proto signal. Phase differences were calculated as the phase of STN minus the phase of Proto at the dominant frequency and expressed in degrees, wrapped to [-180°, 180°]. For each population, the phase at the dominant frequency (frequency with maximum power in the FFT power spectrum) was extracted from the complex FFT coefficient of the demeaned firing rate.

### Single neuron models

In examining the locking of single neurons to periodic inhibition, we use


$$\begin{array}{c} v^{\prime }=I_{1}-v-I_{inh}(t) \end{array}$$ (2)

for leaky integrate-and-fire (LIF) neurons and


$$\begin{array}{c} v^{\prime }=I_{2}+v^{2}-I_{inh}(t) \end{array}$$ (3)

for quadratic integrate-and-fire (QIF) neurons, where *I*_1_ and *I*_2_ denote the constant input currents for the respective models. We also examine the exponential integrate-and-fire (EIF) model, which provides an intermediate level of biophysical realism between simple integrate-and-fire models and full conductance-based descriptions by incorporating an exponential term that captures the rapid depolarization near spike threshold observed in real neurons:


$$\begin{array}{c} v^{\prime }=-v+I+\Delta_{T}\exp\;\left(\frac{v-v_{T}}{\Delta_{T}}\right)-I_{inh}(t) \end{array}$$ (4)

where $\Delta_{T}$ is the slope factor and $v_{T}$ is the threshold potential governing the exponential term. The exponential term creates a sharp acceleration toward spike threshold that more closely approximates the sodium channel activation underlying action potential initiation than does the spiking behavior of the QIF model.

For all three models, the inhibition function is defined as $I_{inh}(t)=a\cdot H\;\left(\sin\;\left(\frac{2\pi t}{T}\right)\right)$, where *a* is the inhibition amplitude, *H* denotes the Heaviside step function, and *T* is the inhibition period. We implement boundary conditions such that $v(0)=v_{reset}$ and $v(t^{+})=v_{reset}$ when $v(t^{-})=v_{th}$, where $v_{reset}$ is the reset voltage and $v_{th}$ is the firing threshold. Throughout this section we fix $v_{reset}=-0.2$, $v_{th}=1$, and *T* = 2. For LIF neurons we set *I*_1_ = 1.25 and *a* = 0.15; for QIF neurons we set *I*_2_ = 0.62 and *a* = 0.41; for EIF neurons we set $\Delta_{T}=0.14$, $v_{T}=0.0$, *I* = 0.14, and *a* = 0.21. These parameters are chosen such that both the stable and unstable fixed points of the TTS map are visible within a single period *T* = 2; the robustness of the stable phase preference across broader parameter ranges is demonstrated in Fig 9.

For the steady-state phase heatmaps (Fig 9), we used separate parameter sets for each model, summarized in Table 3, informed by non-dimensionalization of the respective physiological neuron equations defined in the Multi-population basal ganglia model section below. Range columns indicate the sweep boundaries; all other values are fixed across the sweep.

**Table 3:** Parameter sets used for the steady-state phase heatmaps. Range columns indicate the sweep boundaries for each parameter; all other values are fixed across the sweep.

Model	*T*	*v*_th_	*v*_*r*eset_	$\Delta_{T}$, $v_{T}$	*I* range	*a* range
LIF	10	1.0	0	–	[1.005, 1.08]	[0.1, 1.0]
QIF	50	1.0	0	–	[0.003, 0.012]	[0.005, 0.02]
EIF	8	1.0	0.1	0.17, 0.2	[0.06, 0.11]	[0.02, 0.1]

### Time-to-spike function and map

We define a map, which we call the time-to-spike map [45], that we will use to analyze phase locking of a single neuron to a periodic inhibitory signal. Suppose that square pulses of inhibition are delivered with period *T*, with inhibition on and off for equal times *T*/2 within each cycle. We define $t_{n}^{in}\in [-T/4,3T/4)$ based on the timing of the *n*-th firing event relative to the inhibitory signal. Specifically, let $t_{n}^{f}$ be the n-th firing time. Let $t_{n}^{mid,1}$ and $t_{n}^{mid,2}$ denote the midpoint of the inhibition-on epoch before and after the *n*-th spike respectively. We have


$$\begin{array}{c} t_{n}^{in}=\left\{ \begin{array}{cc} t_{n}^{mid,1}-t_{n}^{f}\in [-T/4,0] & \text{for }t_{n}^{mid,1}\leq t_{n}^{f}\leq t_{n}^{mid,1}+T/4,\\ t_{n}^{mid,2}-t_{n}^{f}\in [0,3T/4) & \text{for }t_{n}^{mid,1}+T/4<t_{n}^{f}\leq t_{n}^{mid,2}. \end{array}\right. \end{array}$$ (5)

**Fig 1:**
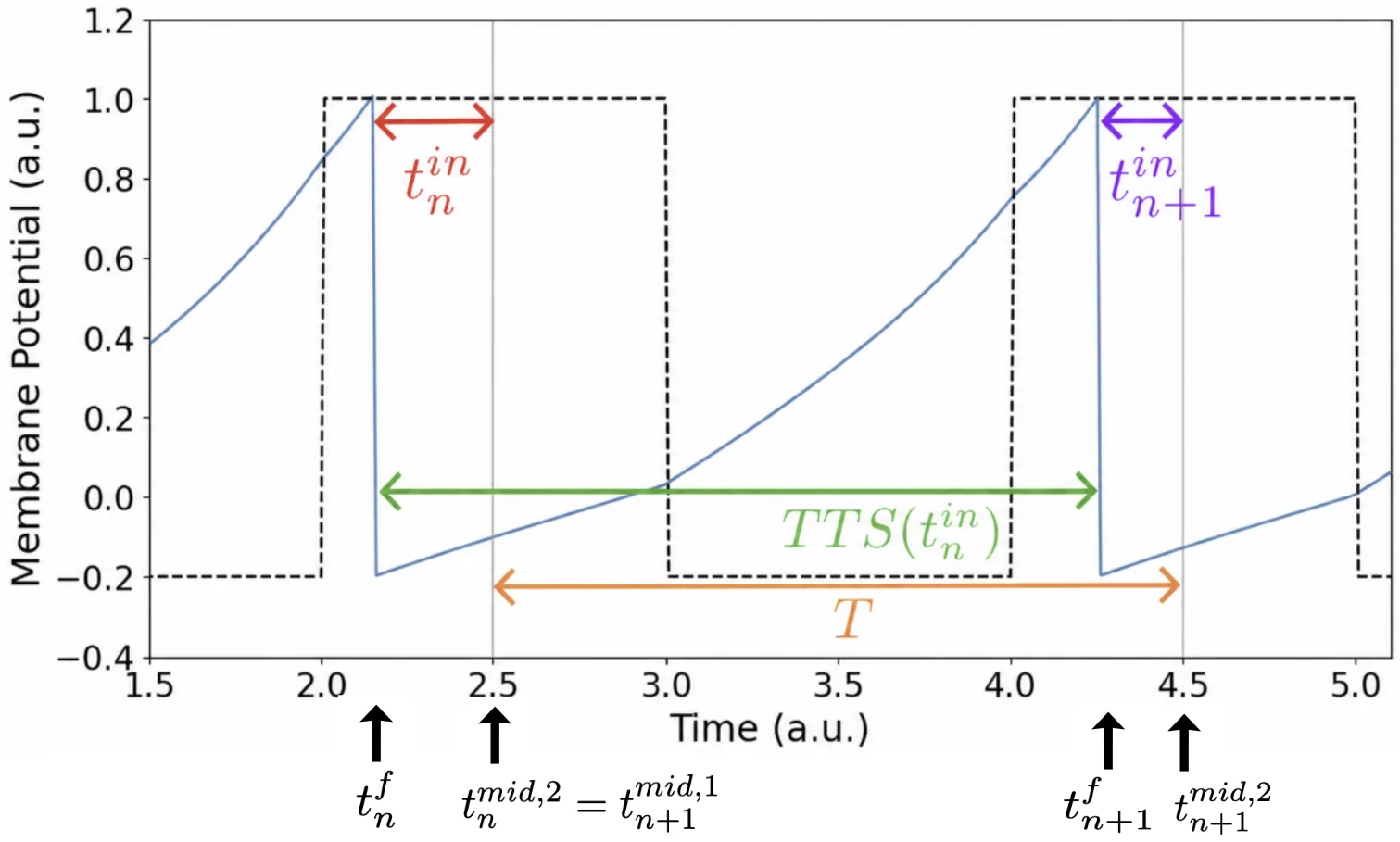
Schematic illustration of the time-to-spike (TTS) mapping. Upward arrows below the time axis mark the firing times $t_{n}^{f}$ and $t_{n+1}^{f}$, as well as the inhibition-on epoch midpoints: $t_{n}^{mid,2}=t_{n+1}^{mid,1}$, the midpoint shared between the epochs immediately after the *n*-th spike and before the (*n* + 1)-th spike, and $t_{n+1}^{mid,2}$, the midpoint of the epoch immediately after the (*n* + 1)-th spike. The diagram shows the relationship between consecutive firing events and inhibitory periods. The time difference between the *n*-th firing event and the center of the subsequent inhibition-on period, $t_{n}^{in}$, evolves to $t_{n+1}^{in}$ through the TTS function, which is the time between the *n*-th and *n* + 1-st firing events. *T* is the period of inhibition.

We also define a time-to-spike function, denoted as $TTS(t_{n}^{in})$, by integrating the neuronal model $v^{\prime }=f(v)-I_{inh}(t)$ from the time of the *n*-th spike, with $v=v_{reset}$, to the time of the (*n* + 1)-st spike, with $v=v_{th}$. Now, to analyze the evolution of phase, we derive a map for $t_{n}^{in}$. By definition, $t_{n+1}^{in}$ represents the time difference between the (*n* + 1)-st firing event and the center of the corresponding inhibition-on period, defined analogously to $t_{n}^{in}$ via equation (5) with updated subscripts. We can also express the map for $t_{n}^{in}$ mathematically using $TTS(t_{n}^{in})$, as illustrated schematically for one case in Fig 1, as


$$\begin{array}{c} t_{n+1}^{in}=\left\{ \begin{array}{cc} t_{n}^{in}-TTS(t_{n}^{in}) & \text{for }\;0\leq TTS(t_{n}^{in})\leq t_{n}^{in}+T/4,\\ T+t_{n}^{in}-TTS(t_{n}^{in}) & \text{for }\;t_{n}^{in}+T/4<TTS(t_{n}^{in})\leq 5T/4+t_{n}^{in}. \end{array}\right. \end{array}$$ (6)

Note that this formulation is easily generalized to cases where there are multiple periods of inhibition between firing events, with *TTS* larger than equation (6) allows, but we do not pursue that here.

### Multi-population basal ganglia model

We assembled collections of spiking neurons into a multi-population basal ganglia model. Individual neurons were represented by LIF, QIF, or EIF equations, specified as follows to incorporate synaptic interactions.

#### Leaky integrate-and-fire (LIF) model.

The membrane potential *V* of the LIF neuron evolves according to:


$$\begin{array}{c} \tau_{m}\frac{dV}{dt}=-(V-E_{L})+I_{\text{syn}}+I_{\text{ext}} \end{array}$$ (7)

where $\tau_{m}$ is the membrane time constant, $E_{L}$ is the leakage potential, *I*_syn_ is the synaptic current, and *I*_ext_ is the external current. When *V* reaches *V*_peak_, the neuron fires a spike, and *V* is reset to *V*_reset_.

#### Quadratic integrate-and-fire (QIF) model.

The membrane potential of the QIF neuron satisfies:


$$\begin{array}{c} \tau_{m}\frac{dV}{dt}=k(V-E_{L})(V-V_{th})+I_{\text{syn}}+I_{\text{ext}} \end{array}$$ (8)

where *k* = 1 is a scaling factor and $V_{th}=-54$ mV is a non-threshold parameter governing the parabolic shape of the voltage dynamics. When *V* reaches *V*_peak_, the neuron fires a spike, and *V* is reset to *V*_reset_.

#### Exponential integrate-and-fire (EIF) model.

The membrane potential of the EIF model obeys the equation:


$$\begin{array}{c} \tau_{m}\frac{dV}{dt}=-(V-E_{L})+\Delta_{T}\exp\;\left(\frac{V-V_{th}}{\Delta_{T}}\right)+I_{\text{syn}}+I_{\text{ext}} \end{array}$$ (9)

where $\Delta_{T}=16.2$ mV is the spike slope factor and $V_{th}=-64$ mV is the threshold potential governing the exponential term. When *V* reaches *V*_peak_, the neuron fires a spike, and *V* is reset to *V*_reset_.

To investigate how non-identical neurons affect population dynamics, we introduced heterogeneous external input currents for a parameter sweep analysis (Fig 13). Each neuron *i* received an external input drawn from a Gaussian distribution:


$$\begin{array}{c} I_{\text{ext},i}\sim 𝒩(\mu_{I},\sigma_{I}^{2}) \end{array}$$ (10)

where $\mu_{I}$ is the mean input current for the population and $\sigma_{I}=0.1\mu_{I}$ for LIF neurons and $\sigma_{I}=0.01\mu_{I}$ for QIF neurons. All other simulations used homogeneous populations where each neuron within a population received identical external input currents. This heterogeneity allowed us to examine how neuronal variability within populations influences the phase coupling between STN and Proto and the resulting beta oscillation dynamics.

Synaptic interactions were modeled using exponential synapses with a time constant τ:


$$\begin{array}{c} \tau \frac{ds_{i}}{dt}=-s_{i}+\sum_{j}w_{ij}\sum_{f}\delta (t-t_{j}^{f}-d_{ij}) \end{array}$$ (11)

where $s_{i}$ is the synaptic gating variable of the neuron *i*, $w_{ij}$ is the connection weight from neuron *j* to neuron *i*, $t_{j}^{f}$ is the firing time of the presynaptic neuron *j*, and $d_{ij}$ is the synaptic delay in the connection from neuron *j* to neuron *i*. The synaptic input is given by $I_{\text{syn},i}=gs_{i}$, where *g* is the maximal coupling strength.

Table 4 shows the parameters for each neuronal population, and Table 5 shows the connection parameters between populations. Parameters are adapted from [39,40]. Negative strength values indicate inhibitory connections, while positive values indicate excitatory connections.

**Table 4:** Population parameters.

Population	Model	Neurons	$\tau_{m}$ (ms)	*V*_peak_ (mV)	$E_{L}$ (mV)	*V*_reset_ (mV)	*I*_ext_ (pA)
D2	LIF	6000	4.9	-50.0	-76.8	-76.8	24.8
Proto	LIF	780	12.9	-54.8	-65.0	-65.0	16.0
FSI	LIF	420	3.1	-52.4	-78.2	-78.2	25.5
STN (LIF)	LIF	408	5.1	-50.8	-59.0	-59.0	9.1
STN (QIF)	QIF	408	20.0	15.0	-70.0	-70.0	66.0
STN (EIF)	EIF	408	6.0	15.0	-80.2	-70.0	2.5

**Table 5:** Connection parameters.

Source	Target	Probability	Delay (ms)	*g* (mV)	τ (ms)
D2	Proto	0.0833	7.0	-0.460	5.5
FSI	D2	0.0524	1.7	-1.216	10.0
Proto	FSI	0.0128	7.0	-1.170	5.5
Proto	STN	0.0385	1.0	-0.096	8.0
STN	Proto	0.0735	2.0	0.061	10.0

We analyzed network dynamics using the following measures:

#### Population firing rate.

For each population, the instantaneous firing rate was calculated by concatenating all spike times within the population and binning them with a time window of 2 ms. The firing rate was computed as the number of spikes per bin divided by the bin width and the number of neurons in the population, expressed in Hz.

#### Power spectral density.

Power spectra were computed using Welch’s method (scipy.signal.welch) with a segment length of 256 samples and default overlap. The sampling frequency was determined from the bin width (500 Hz for 2 ms bins).

#### Beta power quantification.

Beta power was defined as the average power spectral density within the beta frequency band (12.5-30 Hz). For each simulation, beta power was calculated from the population firing rate time series using scipy.signal.welch with nperseg = 256. An initial transient period of 1000 ms was excluded to ensure steady-state dynamics.

#### Average beta frequency.

The average beta frequency of each population was computed as the power-weighted mean frequency (spectral centroid) within the beta band (12.5–30 Hz):


$$\begin{array}{c} {\bar{f}}_{\beta }=\frac{\sum_{i}f_{i}\cdot P_{i}}{\sum_{i}P_{i}} \end{array}$$ (12)

where $f_{i}$ are the discrete frequency bins and $P_{i}$ the corresponding power spectral density estimates obtained via Welch’s method (scipy.signal.welch, nperseg = 256). An initial transient period of 1000 ms was excluded, consistent with the beta power quantification above.

#### Spectrograms.

Time-frequency analysis was performed using scipy.signal.spectrogram with a segment length of 256 samples and overlap of 128 samples. The firing rate signal was demeaned by subtracting the mean before spectrogram computation to remove the DC component.

## Results

### Effect of phase relationship on oscillation amplitude in a rate model

**Fig 2:**
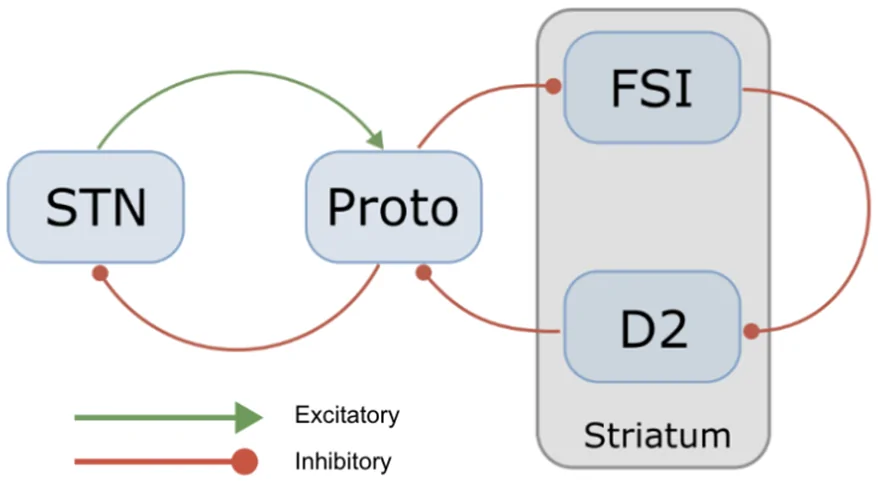
Schematic diagram of the basal ganglia circuit model showing four neuronal populations: subthalamic nucleus (STN), external globus pallidus prototypical neurons (Proto), striatal D2-type spiny projection neurons (D2), and striatal fast-spiking interneurons (FSI). Red arrows indicate inhibitory connections and the green arrow indicates an excitatory connection.

To investigate how STN neuron dynamics affects beta oscillations in the broader basal ganglia network, we developed simplified rate models and spiking networks adapted from more complex, previously published computational models [39,40]; see the previous section for details. Our network architecture (Fig 2) incorporates both the pallidostriatal and subthalamopallidal circuits, including four key neuronal populations: the excitatory subthalamic nucleus (STN) and the inhibitory external globus pallidus prototypical neurons (Proto), striatal D2-type spiny projection neurons (D2), and striatal fast-spiking interneurons (FSI). We focus on the subthalamopallidal (STN↔Proto) and pallidostriatal (D2→Proto→FSI→D2) loops, excluding arkypallidal neurons. This simplification allows us to isolate the core contradiction between recent studies: [39] showed that STN is necessary for beta oscillations generated by these coupled loops, while [40] found that STN suppresses them. Although arkypallidal projections can contribute to beta dynamics, they are not necessary for the fundamental disagreement we aim to resolve regarding how STN-Proto interactions modulate oscillations of pallidostriatal origin.

Recurrent connections within the inhibitory populations were omitted for simplicity, while experimental evidence suggests an abscence of recurrent excitatory connections within the STN population. We initialize the model such that the pallidostriatal loop (D2→Proto→FSI→D2) generates baseline beta oscillations, while the subthalamopallidal circuit (STN↔Proto), which does not oscillate on its own, provides modulatory input. In this section, we will show how the oscillation amplitude in this coupled network depends on the phase relationship between STN and Proto firing rates.

**Fig 3:**
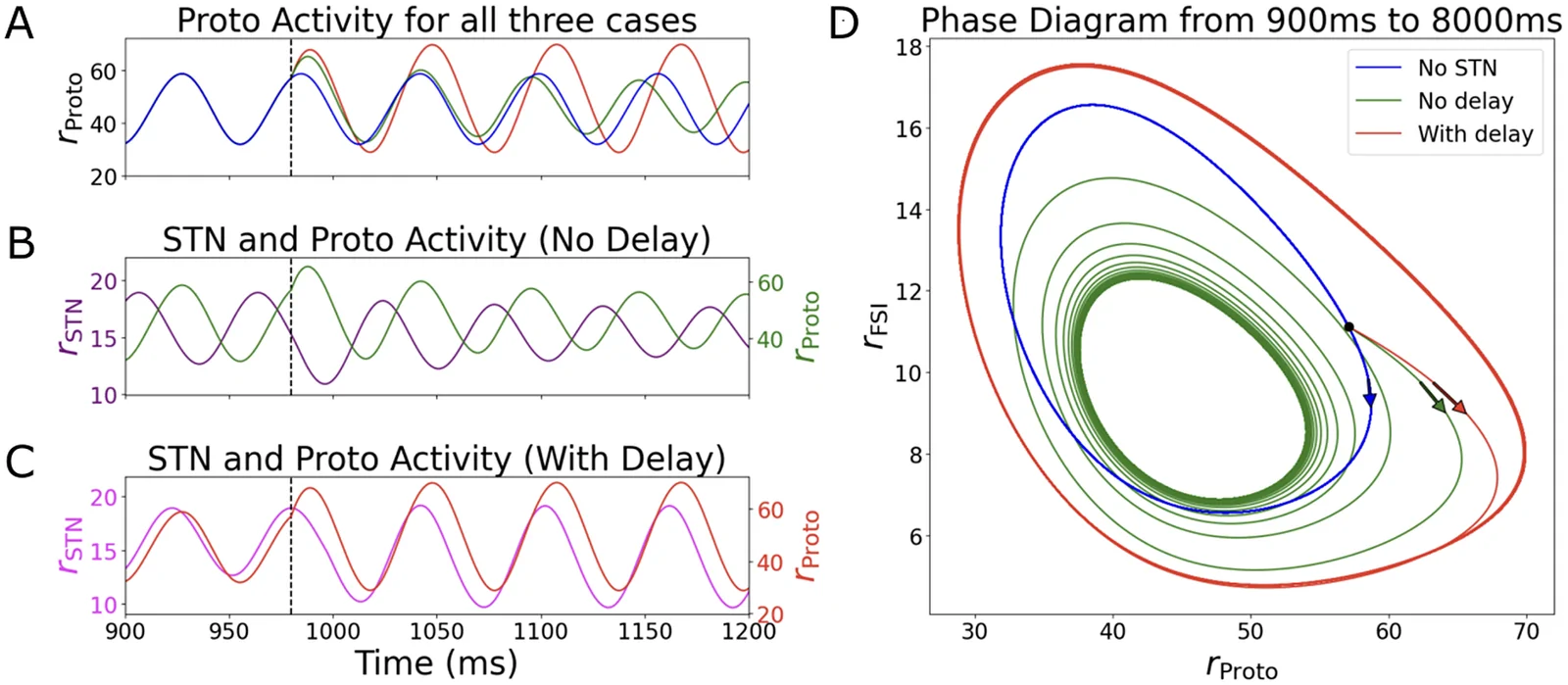
Rate model simulations demonstrating how STN-Proto phase relationships modulate oscillation amplitude. In the context of ongoing pallidostriatal oscillations and input from Proto to STN, the STN to Proto connection is activated at *t* = 975 ms. (A) Proto population activity under three conditions: baseline without STN (blue), STN input with no delay (green), and STN input with 16 ms delay (red; in this case, the input to STN is based on Proto activity 16 ms earlier). (B) Anti-phase relationship of STN and Proto firing rates in the no-delay condition. (C) In-phase STN-Proto phase relationship with 16 ms delay. (D) Trajectories in state space (900–8000 ms) illustrating the mechanistic basis for the amplitude effects: anti-phase excitation arrives during Proto activity troughs creating smaller orbits (green), while in-phase excitation aligns with rising Proto activity producing larger orbits (red). Arrows highlight divergence of trajectory paths following STN activation.

To investigate how phase relationships between STN and Proto populations affect oscillation amplitude, we first examine some specific example cases in a simplified firing rate model. We manipulate the delay from Proto to STN to control their phase relationship. In Fig 3, the Proto-FSI-D2 circuit is configured to generate a stable periodic oscillation. The Proto population drives the STN, and the STN to Proto connection is activated at t = 975 ms to examine how STN input modulates the ongoing oscillation. The results of 3 cases are shown: without STN, with STN but no delay, and with both STN and delay in the Proto-to-STN connection. Without delay, the two populations’ firing rates show an anti-phase relationship (Fig 3B). As we add sufficient delay the two populations switch to an in-phase relationship (Fig 3C, 16 ms delay). Notably, the introduction of STN with no delay (Fig 3A, green curve) decreases the Proto oscillation amplitude compared to the baseline condition (blue curve), while the addition of delay (red curve) substantially increases the amplitude. These amplitude variations can be understood through the diagram in Fig 3D, which illustrates the trajectory of oscillations in the state space. For the no-delay case, the STN excitation arrives during the Proto activity trough, reducing its depth and dampening the overall oscillatory behavior. Conversely, in the with-delay condition, the excitation aligns with the rising phase of Proto activity, reinforcing and amplifying the oscillations.

**Fig 4:**
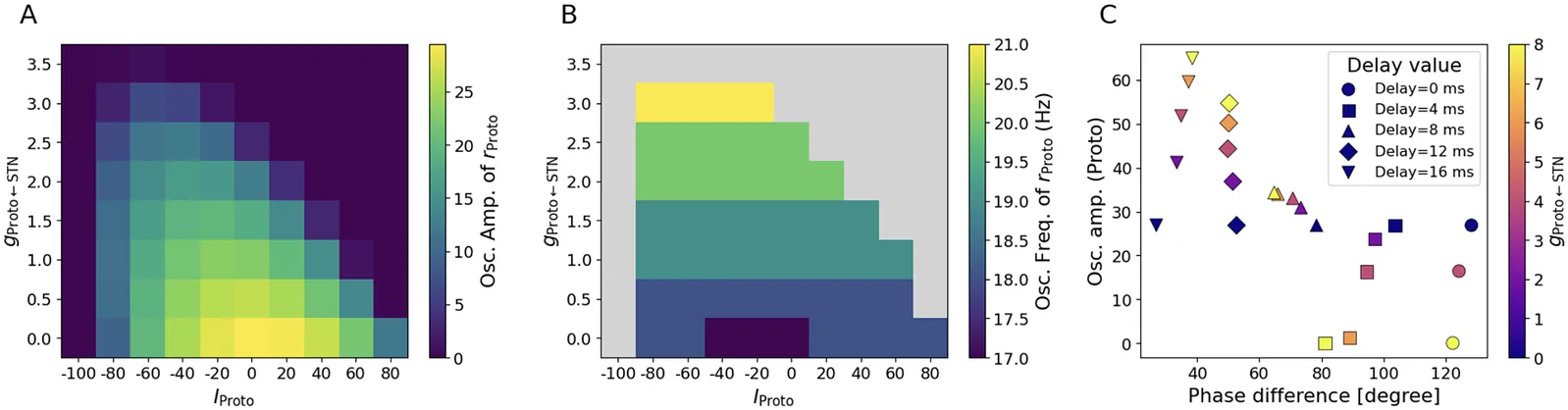
Parameter exploration of factors affecting beta oscillation amplitude in the reciprocally coupled STN-Proto circuit. (A) Heat map showing Proto oscillation amplitude (color) as a function of STN to Proto connectivity strength ($g_{\text{Proto}\leftarrow \text{STN}}$) and drive to Proto (*I*_Proto_). The amplitude decreases with increasing connectivity strength across the full range of drive values, including regions between the two Hopf bifurcations, demonstrating that the amplitude reduction is not attributable to the second Hopf bifurcation. (B) Heat map showing the oscillation frequency of Proto (color) across the same parameter space. The oscillation frequency remains within the beta band (~17−21 Hz) across all parameter combinations that exhibit appreciable oscillations. Grey regions indicate parameter combinations where oscillation amplitude falls below 1 and are therefore excluded. (C) Scatter plot depicting the relationship between phase difference and oscillation amplitude across different Proto to STN delay values ($d_{\text{STN}\leftarrow \text{Proto}}$). Each point represents a different connectivity strength ($g_{\text{Proto}\leftarrow \text{STN}}$, color-coded), with symbols indicating delay values (circles: 0 ms, squares: 4 ms, triangles: 8 ms, diamonds: 12 ms, inverted triangles: 16 ms). The plot reveals that shorter delays produce larger phase differences (anti-phase, 90 + degrees) with lower amplitudes, while longer delays generate smaller phase differences (in-phase, ~20-60 degrees) with higher amplitudes.

To systematically investigate how the phase relationship between STN and Proto populations modulates beta oscillation amplitude in Proto, we conducted a parameter exploration study. Fig 4 presents the results of this analysis. Fig 4A shows Proto oscillation amplitude as a function of the drive to Proto (*I*_Proto_) and the STN to Proto connection strength ($g_{\text{Proto}\leftarrow \text{STN}}$) without any delay, with the Proto-STN connection strength held fixed. Varying *I*_Proto_ for fixed $g_{\text{Proto}\leftarrow \text{STN}}$ below approximately 3.5 reveals two presumed Andronov-Hopf (AH) bifurcations, as evidenced by the emergence and subsequent disappearance of oscillations along horizontal sections of the heatmap. However, the vertical patterns highlight a key difference: for fixed *I*_Proto_ values in the range of −60 to −20, oscillation amplitude decreases monotonically as $g_{\text{Proto}\leftarrow \text{STN}}$ increases. Notably, increasing $g_{\text{Proto}\leftarrow \text{STN}}$ increases the excitatory drive to Proto, but unlike increasing *I*_Proto_, it does not cause an initial increase in oscillation amplitude before the decrease. This confirms that the decrease in amplitude with small $g_{\text{Proto}\leftarrow \text{STN}}$ is not caused by the occurrence of a second Hopf bifurcation. Fig 4B shows the oscillation frequency of Proto across the same parameter space. The frequency remains within the beta band (~17−21 Hz) throughout all parameter combinations that exhibit appreciable oscillations (amplitude ≥1).

Fig 4C illustrates the relationship between phase difference and oscillation amplitude across different delay and connectivity conditions. The scatter plot reveals a clear inverse relationship: as phase differences decrease (indicating a shift toward in-phase dynamics), oscillation amplitudes increase substantially. At short Proto-to-STN delays (0–4 ms, circles and squares), the STN and Proto populations exhibit large phase differences of approximately 100–120 degrees, corresponding to anti-phase relationships that suppress oscillatory activity and result in low amplitudes (typically below 30). In this case, larger $g_{\text{Proto}\leftarrow \text{STN}}$ values lead to reduced oscillation amplitudes. Conversely, at longer delays (12–16 ms, diamonds and inverted triangles), phase differences decrease to 20–60 degrees, indicating in-phase relationships that enhance oscillatory activity and produce amplitudes reaching 50–60. Here, greater $g_{\text{Proto}\leftarrow \text{STN}}$ strengths yield enhanced oscillation amplitudes. The intermediate delay condition (8 ms, triangles) shows a transitional behavior with moderate phase differences around 60–80 degrees and intermediate amplitudes, with less dependence on $g_{\text{Proto}\leftarrow \text{STN}}$.

Together, these results provide a comprehensive view of how both connectivity strength and relative timing of STN and Proto oscillations, dictated by the delay in input from STN to Proto, contribute to the generation and modulation of beta oscillations in the basal ganglia circuit.

### Single neuron dynamics and phase relationships under periodic inhibition

**Fig 5:**
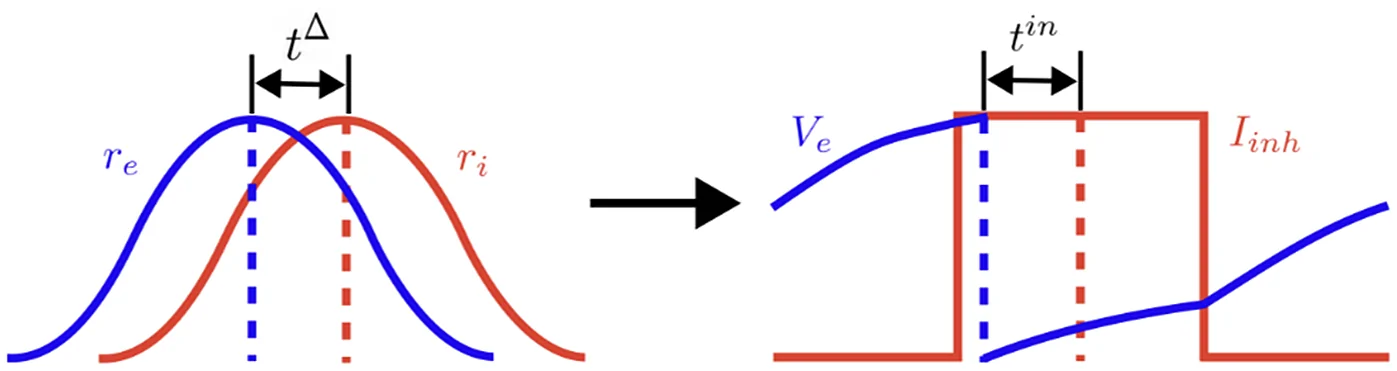
Schematic illustration of a simplified modeling approach for analyzing phase relationships between excitatory and inhibitory populations. The figure shows the transition from the definition of $t^{\Delta }$ as the time difference between the peaks of excitatory (E) and inhibitory (I) population firing rates (left) to the simplified single neuron model where $t^{in}$ is redefined as the temporal offset between the excitatory neuron’s spike times, where its voltage $V_{e}$ reaches a maximum and is reset, and the mid-point of each pulse of square-wave inhibition that the excitatory neuron receives (right). This simplification preserves the essential phase locking effect while allowing for an analytical treatment of the underlying mechanisms.

Having established the influence of phase relationship on oscillation amplitude, we next asked what could cause different phase relationships to materialize. We hypothesized that within a fixed circuit structure, the choice of neuronal model used would determine the phase differences that emerge. Our goal in this section is to understand how different models could yield distinct phase relationships between coupled excitatory and inhibitory populations. We define $t^{\Delta }$ to be the difference in times of firing rate peaks of the two populations, as depicted by the first panel of Fig 5. Initially, we consider a highly simplified scenario in which we replace the inhibitory population and its firing rate with a periodic train of square pulses of inhibition, with inhibition on for half of each period and off for the other half as discussed in *Models and Methods*, and replace the excitatory population and its firing rate with the voltage and spike times of a single excitatory neuron. In this setting, $t^{in}$ is defined to be the time difference between the spike of the excitatory neuron and the mid-point of the time that its inhibitory input is on within each cycle, as depicted by the second panel of Fig 5. We will perform some computations and analysis of phase locking in this simplified setting, and later we will show with computational results that networks of spiking neurons exhibit similar properties to the simplified system.

#### Examples of phase relationships.

We next present examples illustrating different phase relationships between neuronal firing and inhibitory input in QIF and LIF neurons (see Methods section). We demonstrate a fundamental difference in phase preferences: QIF neurons tend to fire during inhibition periods (in-phase), while LIF neurons preferentially fire when inhibition is absent (anti-phase).

**Fig 6:**
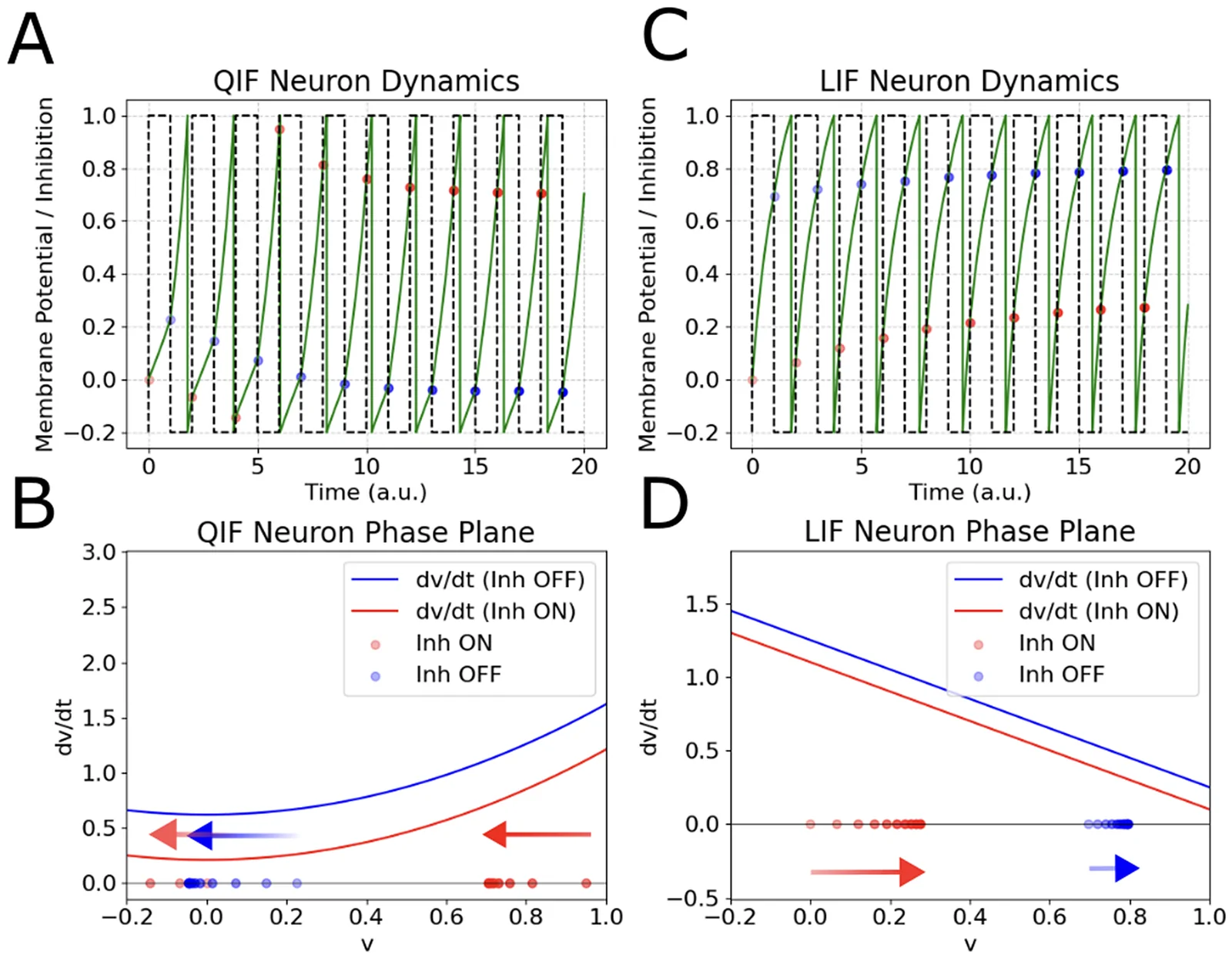
Comparison of QIF and LIF neuron dynamics under square-wave inhibitory forcing. (A) Membrane potential dynamics of a QIF neuron. Red dots indicate moments when inhibition turns ON, blue dots when inhibition turns OFF. (B) Phase plane representation of the QIF neuron, with membrane potential (*v*) on the x-axis and *dv*/*dt* on the y-axis. Dots correspond to the same time points shown in panel A, with matching transparency indicating simultaneous moments across the two panels. Lighter dots correspond to earlier times, and the arrows indicate the chronological order of the blue and red dots, respectively. (C) and (D) are similar figures for an LIF neuron. Note how QIF neurons preferentially fire during inhibition (in-phase relationship), while LIF neurons fire predominantly when inhibition is absent (anti-phase relationship).

Fig 6 provides a visual representation of these contrasting behaviors. In the QIF model (panels A and B), the membrane potential (green curve) evolves so that, after a few transient cycles, it reaches the firing threshold (*v* = 1) during periods of inhibition (black dashed). Following each threshold crossing, the potential is reset to $v_{reset}=-0.2$. Conversely, in the LIF model (panels C and D), threshold crossings occur during periods when inhibition is off. The phase plane representations in panels B and D reveal the underlying dynamical structure responsible for these different behaviors, which will be analyzed formally in subsequent sections. We have chosen parameters so that the neurons are phase-locked to the periodic inhibition, and they converge to steady-state periodic behavior over time.

In all four panels of Fig 6, red and blue dots indicate the neuron’s voltage when inhibition turns on and off, respectively. The transparency of the dots, mostly visible in panels B and D, reflects their temporal occurrence, with higher transparency (paler) dots indicating earlier time points in the simulation. For QIF neurons, the voltage is typically low (near 0) when inhibition is off and high when inhibition is on. LIF neurons exhibit the opposite pattern. The arrows in panels B and D illustrate the convergence pattern of these voltage states toward their steady-state values. These observations provide mechanistic insight into why the two neuron types exhibit different phase relationships. QIF neurons have a saddle-node bifurcation giving rise to two low-voltage equilibria as their inhibition level increases. Whether inhibition is on or off, *dv*/*dt* is small near the “ghost” of these equilibria, but this effect is especially strong with inhibition present. Thus, if the neuron fires with inhibition off on one cycle, then its voltage stays close to the reset value throughout the inhibition-on period and then does not have enough time to evolve up to the firing threshold when inhibition is off, resulting in an inhibition-on spike on the next cycle. From another perspective (Fig 6B), the voltage levels at which inhibition turns on and off show phase precession until they stabilize with low (high) voltages when inhibition turns off (on). In contrast, in LIF neurons, the linear form of the *v*-nullcline allows voltage to rise significantly during inhibition yet slows it near threshold, thereby suppressing firing. When inhibition is removed, the increase in *dv*/*dt* allows a faster increase in *v* that facilitates threshold crossing. In this case, voltages when inhibition turns on and off show phase advances and stabilize with high (low) voltages when inhibition turns off (on).

We will next analyze these effects using the *time-to-spike* (TTS) function to provide a mathematical proof demonstrating why in-phase solutions cannot be stable for LIF neurons and confirming the existence of stable in-phase solutions for QIF neurons.

#### Time-to-spike analysis: Fixed points and phase relationships.

We defined a time-to-spike function (6) [45] that serves as a map from the time when a periodically driven neuron spikes on one input cycle, relative to a reference time defined based on the drive signal, to the corresponding relative time on the next input cycle (see Methods). An anti-phase steady state corresponds to $T/4<t_{ss}^{in}<3T/4$, where neurons fire during the inhibition-off period, while an in-phase solution corresponds to $-T/4<t_{ss}^{in}<T/4$, where neurons fire during inhibition. To solve for the fixed point $t_{ss}^{in}$, first note that a fixed point can only occur for the second case of equation (6), since $TTS(t_{n}^{in})>0$. We substitute $t_{n+1}^{in}=t_{n}^{in}=t_{ss}^{in}$ into this expression to obtain $TTS(t_{ss}^{in})=T$. For the fixed point to be stable, we need the derivative on the right-hand side to be between −1 and 1. Hence, we require $0<TTS^{\prime }(t_{ss}^{in})<2$.

**Fig 7:**
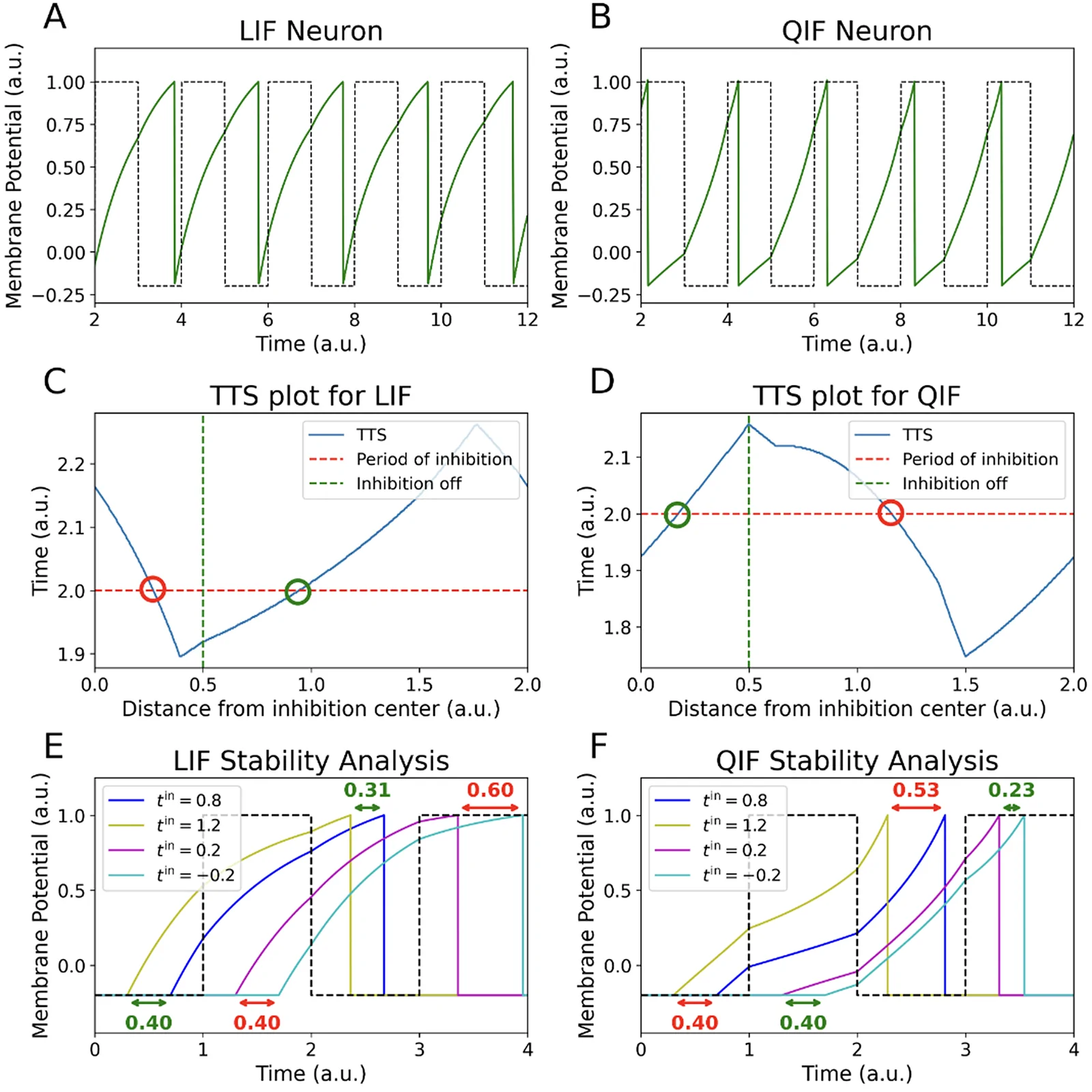
Numerics of Time-To-Spike (TTS) functions and fixed points for LIF and QIF neurons with a period of 2 ms. (A) Phase relationship in LIF neurons showing predominant firing during inhibition-off periods (anti-phase). (B) Phase relationship in QIF neurons showing predominant firing during inhibition-on periods (in-phase). (C) TTS function for LIF neurons displaying a stable anti-phase fixed point ($t_{LIF}^{AP}$, green circle) and an unstable in-phase fixed point ($t_{LIF}^{IP}$, red circle). (D) TTS function for QIF neurons with opposite fixed point stability: a stable in-phase fixed point ($t_{QIF}^{IP}$, green circle) and an unstable anti-phase fixed point ($t_{QIF}^{AP}$, red circle). (E) Single-step TTS mapping for LIF neurons demonstrating regions of reset positions (with corresponding timings $t^{in}$ relative to the midpoint of inhibition as shown in the legend) that lead to contraction (green) and dilation (red), illustrating the stability of the anti-phase fixed point $t_{LIF}^{AP}$ and the instability of the in-phase fixed point $t_{LIF}^{IP}$. (F) Single-step TTS mapping for QIF neurons demonstrating analogous regions with reversed roles: contraction (green) near the in-phase fixed point $t_{QIF}^{IP}$ and dilation (red) near the anti-phase fixed point $t_{QIF}^{AP}$, consistent with the opposite stability properties of QIF neurons.

Fig 7 illustrates the numerically computed TTS functions and their corresponding fixed points for both LIF and QIF neurons with a period of *T* = 2 ms, set by the period of the inhibition function. For LIF neurons (panels A-B), our numerics reveals a stable fixed point, which we will denote as $t_{LIF}^{AP}$, with a distance above *T*/4 = 1/2 from the inhibition center (green circle) corresponding to anti-phase firing during inhibition-off periods. Additionally, an unstable fixed point $t_{LIF}^{IP}$ (red circle) exists at a distance less than *T*/4 from the inhibition center, corresponding to in-phase firing.

Conversely, QIF neurons (panels C-D) exhibit the opposite behavior, with a stable fixed point, $t_{QIF}^{IP}$, corresponding to in-phase firing and an unstable fixed point, $t_{QIF}^{AP}$, corresponding to anti-phase firing. This fundamental difference in dynamics agrees with and provides mathematical support for the contrasting phase relationships observed in Fig 6.

The stability properties of these fixed points can be understood from the slope of the TTS function at each intersection with the $TTS(t^{in})=T$ line. Fig 7E demonstrates a contraction (green region) between trajectories that occurs on an interval of initial reset times for the LIF model corresponding to different timings relative to the ongoing inhibitory signal, which constrains the slope of the TTS function such that $0<TTS^{\prime }(t_{LIF}^{AP})<2$ and hence leads to the stability of anti-phase solutions. Conversely, the red region represents a dilation giving rise to the slope $TTS^{\prime }(t_{LIF}^{IP})<0$, corresponding to the instability of in-phase solutions. Analogous regions are shown for the QIF model in Fig 7F. These mathematical properties, formalized in Appendices A and B in S1 Appendix, provide a rigorous foundation for understanding the emergence of different phase relationships in these neuronal models. The return maps of the TTS function, visualizing the convergence of iterates to these fixed points, are shown in Appendix D in S1 Appendix and S2 Fig. In Appendix C in S1 Appendix, including S1 Fig, we present numerical illustrations of these properties.

#### Exponential integrate-and-fire neurons.

Our investigation into how neuronal model choice affects beta oscillation dynamics was originally motivated by the computational study of [39], which demonstrated that dopamine depletion leads to pathological synchronization in the beta band, with their results suggesting that STN activity is essential for beta generation. Notably, their model implemented STN neurons using the exponential integrate-and-fire (EIF) framework rather than the QIF neurons we analyzed in previous sections. To verify that our conclusions regarding phase relationships and beta modulation hold for the original neuronal model used in their study, we examined EIF neuron dynamics under periodic inhibition.

**Fig 8:**
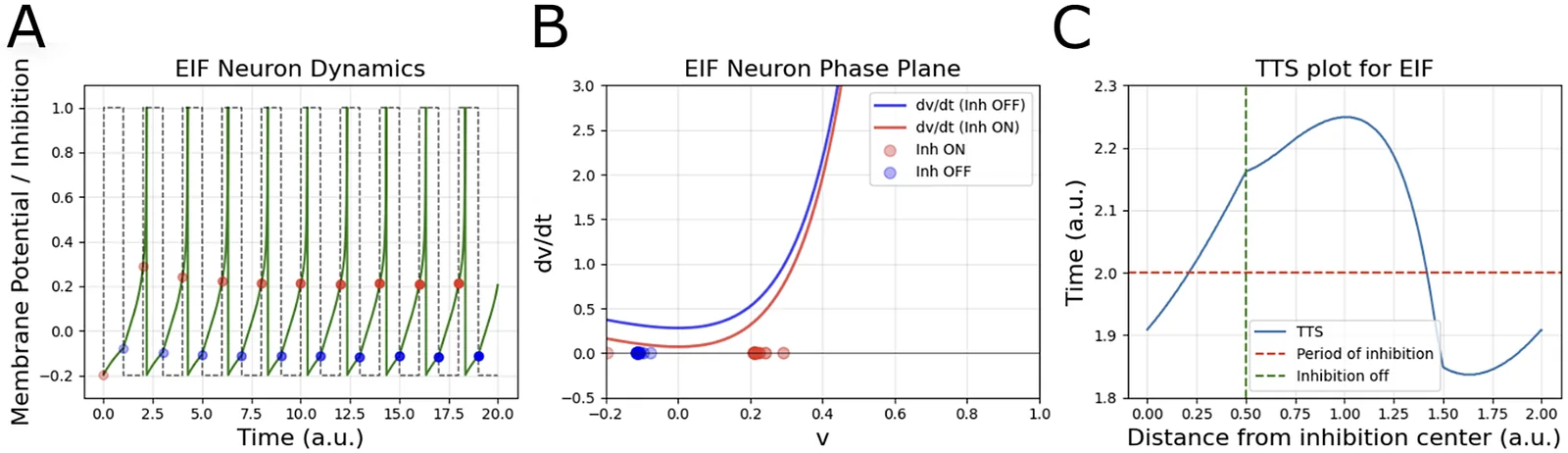
Exponential integrate-and-fire (EIF) neuron dynamics under periodic inhibition demonstrate in-phase firing preference similar to QIF neurons. (A) Membrane potential dynamics showing preferential firing during inhibition-on periods (red dots). (B) Phase plane representation showing rapid acceleration toward threshold during inhibitory periods (red) and low voltages while inhibition is off (blue). (C) Time-to-spike function with a stable (unstable) fixed point corresponding to an in-phase (anti-phase) relationship of neural firing relative to the inhibitory input, similar to patterns observed in Fig 7 for QIF neurons.

Our simulations of EIF neurons under periodic inhibition display striking similarities to QIF neuron behavior, confirming that the conclusions drawn from [39] align with our theoretical framework. Fig 8 demonstrates that EIF neurons exhibit a clear preference for in-phase firing with periodic inhibition. The membrane potential dynamics (Fig 8A) show that spikes consistently occur during inhibition-on periods (red dots), with the neuron maintaining low membrane potentials during inhibition-off periods (blue dots), mirroring the pattern observed in QIF neurons (Fig 6A).

Phase plane analysis (Fig 8B) reveals the mechanistic basis for this in-phase preference. The *dv*/*dt* nullclines for inhibition-on (red) and inhibition-off (blue) conditions show that the exponential term in EIF neurons creates dynamics similar to those observed in QIF neurons (Fig 6B). During inhibition (red), the exponential acceleration still allows the neuron to reach threshold, while during inhibition-off periods (blue), the membrane potential tends to settle at lower values. The time-to-spike function for EIF neurons (Fig 8C) exhibits qualitative agreement with that for QIF neurons (Fig 7D), with a stable fixed point, $t_{EIF}^{IP}<0.5$, where the TTS curve intersects the inhibition period (red dashed line), corresponding to an in-phase solution.

These findings provide direct validation that the results of [39] are consistent with our theoretical predictions. Since EIF neurons exhibit in-phase coupling preferences similar to QIF neurons, STN populations modeled with EIF dynamics would establish in-phase relationships with Proto populations, thereby enhancing beta oscillations generated by the pallidostriatal circuit—exactly as observed in their study.

The similarity between EIF and QIF neuron dynamics in terms of phase relationships strengthens our conclusion that the mathematical structure underlying spike generation, rather than specific biophysical details, determines phase preferences under oscillatory conditions. Both models share the property of rapid, nonlinear approach to threshold, which enables firing during inhibitory periods when the neuron has sufficient momentum to overcome the additional hyperpolarizing drive.

#### Phase relationships across parameter regimes.

**Fig 9:**
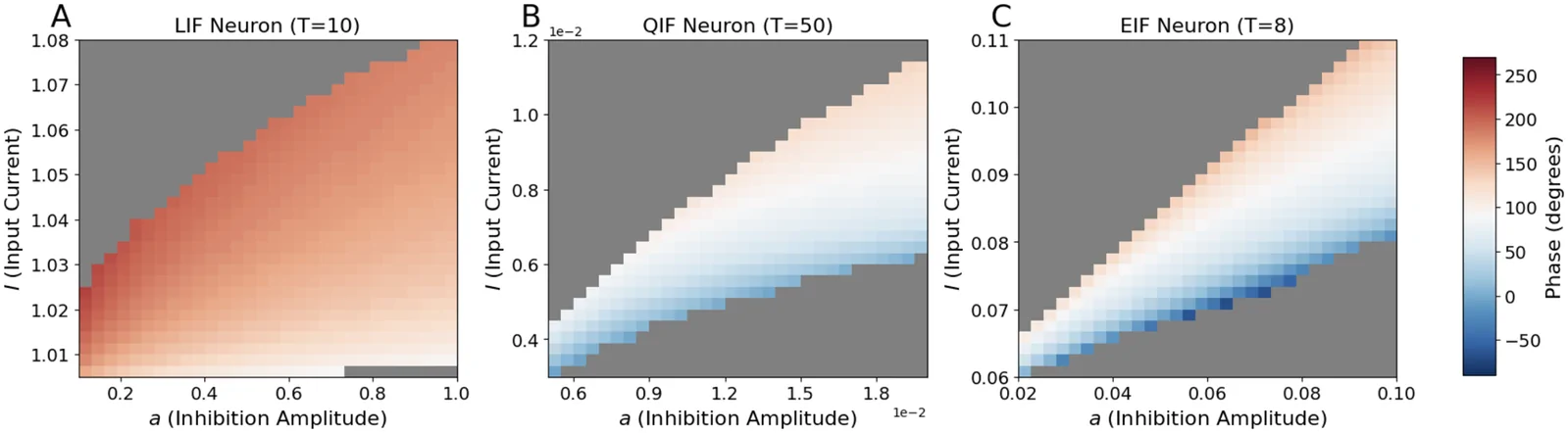
Steady-state phase relationships as a function of excitatory drive *I* and inhibition amplitude *a* for all three neuron models. Parameters for each model are given in Table 3. The colormap is centered at white, corresponding to $90^{\circ }$, with red indicating anti-phase values ($>90^{\circ }$) and blue indicating in-phase values ($<90^{\circ }$). (A) LIF neurons exhibit phase values exclusively in the anti-phase range ($90^{\circ }-270^{\circ }$) across all parameter combinations where phase-locking exists. (B) QIF neurons and (C) EIF neurons both display predominantly in-phase locking (blue) at low inhibition amplitudes; however, as inhibition amplitude *a* increases, anti-phase solutions begin to appear alongside the dominant in-phase locking, expanding the range of accessible phase relationships. Gray regions denote parameter combinations where no stable phase-locked solution exists.

To characterize how the steady-state phase depends on excitatory drive and inhibition amplitude across all three neuron models, we computed the fixed points of the time-to-spike map over a range of physiologically relevant parameters. For each combination of inhibition amplitude *a* and input current *I*, we numerically identified stable fixed points satisfying $TTS(t_{ss}^{in})=T$ with $0<TTS^{\prime }(t_{ss}^{in})<2$, and calculated the corresponding phase as $(t_{ss}^{in}/T)\times 360^{\circ }$. Color-coded regions in Fig 9 are displayed only where stable fixed points exist; white regions indicate parameter combinations where phase-locking is absent.

LIF neurons (Fig 9A) exhibit phase values predominantly ranging from $90^{\circ }$ to $270^{\circ }$, reflecting a consistent anti-phase preference across the parameter space. In contrast, QIF neurons (Fig 9B) show phase values predominantly below $90^{\circ }$, indicating a robust in-phase preference. EIF neurons (Fig 9C) also exhibit in-phase solutions across much of the parameter space, though with greater variability in the phase values observed. The analytical conditions governing the existence and location of these fixed points are derived in the Appendices A and B in S1 Appendix, and validated numerically in S2 Fig. The effect of the input current *I* and oscillation period *T* on the steady-state phase is further illustrated in S3 Fig, which shows that the contrasting phase preferences of LIF and QIF/EIF neurons persist across a wide range of values for these parameters. This robustness is also demonstrated in Appendix E in S1 Appendix, including S4 Fig and S5 Fig, where both the TTS functions and parameter sweeps are reproduced under sinusoidal inhibition.

Together, the single-neuron analysis, parameter sweeps across all three neuron models and mathematical proofs establish a consistent picture: the choice of spike-generation mechanism greatly influences whether a neuron locks in-phase or anti-phase to periodic inhibition. Having characterized this phenomenon at the single-neuron level, we next ask whether these phase preferences persist and translate into functionally meaningful differences in a fully connected, multi-population basal ganglia network of spiking neurons.

### Full neuronal network model

Up to this point, we have run simulations with firing rate models and with a spiking STN model coupled to a periodic inhibitory signal. To conclude our study, we next simulated a multi-population basal ganglia network model composed of spiking neurons with interconnections matching the known neuroanatomy (see Methods). We simulated four key populations: D2-type spiny projection neurons (D2), prototypical neurons in the external globus pallidus (Proto), fast-spiking interneurons (FSI), and subthalamic nucleus neurons (STN). The network topology follows the indirect pathway of the basal ganglia, which is known to be involved in beta oscillation generation.

#### Comparison between single neuron analysis and spiking network dynamics.

**Fig 10:**
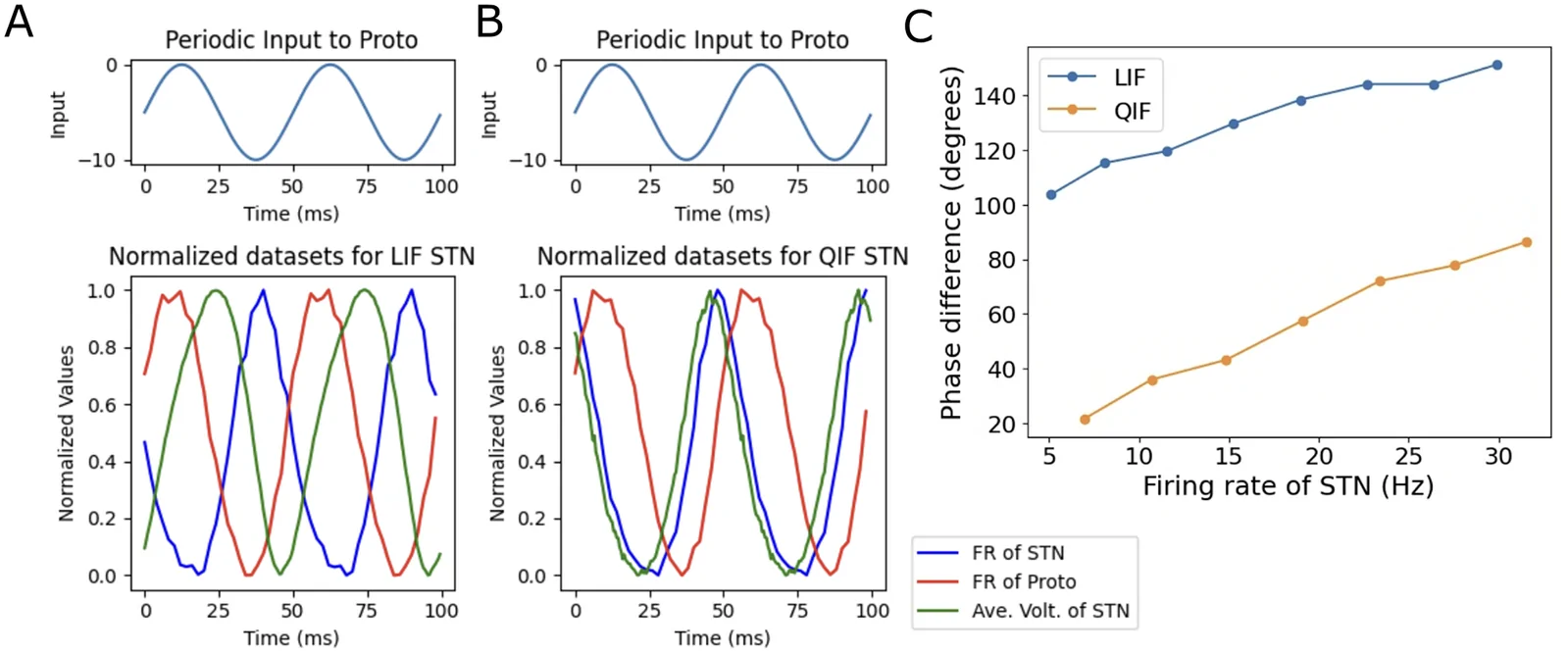
Comparative dynamics of STN-Proto circuits using different neuronal models. (A) Periodic inhibitory input to Proto neurons (top) and anti-phase synchronization between LIF STN and Proto firing rates (bottom), consistent with single neuron studies. (B) Periodic inhibitory input to Proto neurons (top) and in-phase relationship between QIF STN and Proto firing rates (bottom), with STN membrane potential continuing to rise up toward threshold even while Proto firing ramps up. In both (A) and (B), the Proto population consists of LIF model units. (C) Phase difference as a function of firing rate for LIF (blue) and QIF (orange) STN neurons, relative to Proto firing. LIF neurons consistently exhibit larger phase differences (anti-phase preferences), while QIF neurons show smaller phase differences (in-phase tendencies) across the firing rate spectrum.

To validate the biological relevance and broader applicability of our single-neuron analysis, we first implemented spiking network models consisting of interconnected STN and Proto populations, without the striatal elements from Fig 2. Fig 10 presents simulation results from these networks under periodic inhibitory drive to the Proto population, mimicking optogenetic stimulation protocols used in experimental settings [27].

In both network configurations, Proto neurons were modeled as LIF neurons, while STN neurons were implemented as either QIF (Fig 10A) or LIF (Fig 10B) neurons. This allowed us to isolate the effects of excitatory neuron dynamics on network-level phase relationships. The simulations incorporated full connectivity between populations with synaptic delays and conductance-based synapses.

The network-level simulations demonstrate striking concordance with our single-neuron predictions. In the LIF-STN network (Fig 10A), both the STN population firing rate (blue) and average membrane potential (green) exhibit clear anti-phase relationships with the Proto population activity (red), confirming our analytical prediction that LIF neurons tend toward anti-phase locking with periodic inhibition. Notably, the STN membrane potential dynamics (green curve) are consistent with the single-neuron phase plane analysis shown in Fig 6: membrane potentials are low during inhibition-on periods and elevated during inhibition-off periods, matching the voltage state patterns observed in our theoretical analysis.

Conversely, the QIF-STN network (Fig 10B) displays a pronounced in-phase relationship between STN and Proto population activities. Notably, the STN membrane potential rises substantially during periods of Proto inactivity (corresponding to inhibition-off periods in our single-neuron framework), such that STN is able to fire after Proto activity has risen from its trough, demonstrating the mechanistic basis for in-phase preferences in QIF neurons.

To further validate our theoretical predictions beyond the phase-locked regime, we examined how phase relationships vary as a function of STN population firing rate under periodic inhibition (see Methods). Fig 10C presents this analysis for both LIF and QIF STN neurons across a range of firing frequencies. The input current ranges for both model classes were selected to generate firing rates spanning from just above 0 Hz to approximately 30 Hz.

These results demonstrate that the phase preferences we identified persist across different firing rate regimes, even when neurons are not strictly phase-locked to the inhibitory input. LIF neurons (blue curve) consistently exhibit larger phase differences relative to the inhibitory input, confirming their anti-phase preference across the entire firing rate spectrum examined. Conversely, QIF neurons (orange curve) maintain smaller phase differences overall but exhibit a gradual increase in phase difference as firing rate increases. This trend can be explained by the increased input drive: as the excitatory drive to STN neurons is elevated, they eventually begin to fire more readily when inhibition level drops rather than maintaining their preferred in-phase timing.

This analysis extends our findings beyond the idealized phase-locked scenarios and demonstrates that the fundamental phase preferences determined by intrinsic neuronal dynamics remain consistent across physiologically relevant firing rate ranges. The persistence of these phase relationships across different firing frequencies strengthens our conclusion that neuronal model choice critically determines phase dynamics in oscillatory neural circuits.

These simulation results, including both the network dynamics and firing rate analysis, confirm that the phase relationships established in our single-neuron analysis extend robustly to the population level, even in the presence of network-level complexities. The consistency between single-neuron predictions and network behavior underscores the critical role of intrinsic neuronal dynamics in determining population-level synchronization patterns in the STN-Proto circuit.

Moreover, these findings suggest that the divergent phase relationships observed in different experimental and computational studies of parkinsonian oscillations may stem partly from differences in the neuronal models employed, rather than solely from differences in connectivity architectures or external driving forces. This insight provides a new perspective on interpreting seemingly contradictory results in the literature and highlights the importance of carefully considering neuronal model selection when investigating oscillatory phenomena in the basal ganglia.

#### Results on the two-loop spiking network.

Having established that different STN models (LIF vs. QIF) fundamentally determine the phase relationship between STN and Proto populations—with QIF neurons favoring in-phase locking and LIF neurons preferring anti-phase relationships—we now turn to the critical question of how these phase differences impact beta oscillation dynamics in the complete basal ganglia network. Our single-neuron and simplified network analyses provide the mechanistic foundation, but to fully test our hypothesis that contradictory findings in the literature stem from neuronal model choices rather than circuit-level disagreements, we must examine these phase relationships within the broader context of coupled subthalamopallidal and pallidostriatal loops. Thus, we performed comprehensive network simulations to demonstrate that the STN-Proto phase locking tendencies that we have characterized serve as the key determinant of whether STN enhances or suppresses pathological beta oscillations originating in the pallidostriatal system.

**Fig 11:**
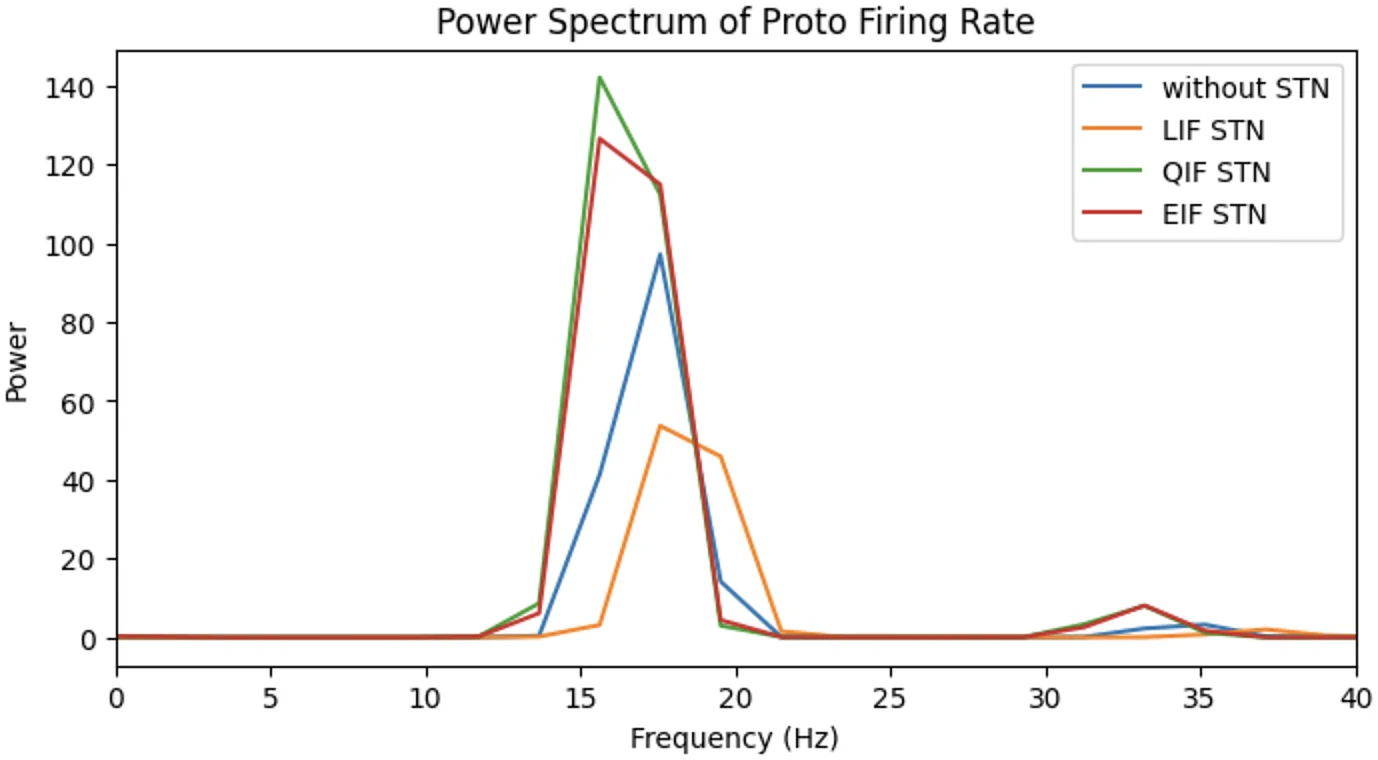
Power spectra of Proto population firing rates across network configurations. The baseline condition without STN (blue) shows beta power generated by the pallidostriatal loop alone. Adding LIF STN neurons (orange) reduces beta power compared to baseline, while both QIF (green) and EIF (red) STN neurons substantially enhance beta oscillations, with a more prominent peak around 15–18 Hz. Thus, single neuron dynamics in STN critically determine whether the subthalamopallidal loop enhances or suppresses beta oscillations generated by the pallidostriatal circuit, and the QIF and EIF models produce qualitatively similar amplification effects.

To investigate how single neuron dynamics influence beta oscillations in the full spiking network, we compared four network configurations: without STN (baseline), and with LIF, QIF, or EIF STN neurons. Fig 11 shows the power spectra of Proto population firing rates for each condition. The baseline network without STN already generates beta oscillations through the pallidostriatal loop, serving as a benchmark for comparison. Adding LIF STN neurons reduces beta power compared to this baseline, while incorporating QIF or EIF STN neurons substantially enhances beta oscillations, producing a more prominent peak around 15–18 Hz. Notably, the QIF and EIF configurations yield qualitatively similar amplification effects, consistent with their shared in-phase firing preference identified in the single-neuron analysis. This result demonstrates that the choice of single neuron model for STN critically determines whether the subthalamopallidal loop enhances or suppresses the beta oscillations generated by the pallidostriatal circuit, consistent with our theoretical predictions that phase relationships between STN and Proto populations are key to oscillation amplitude.

**Fig 12:**
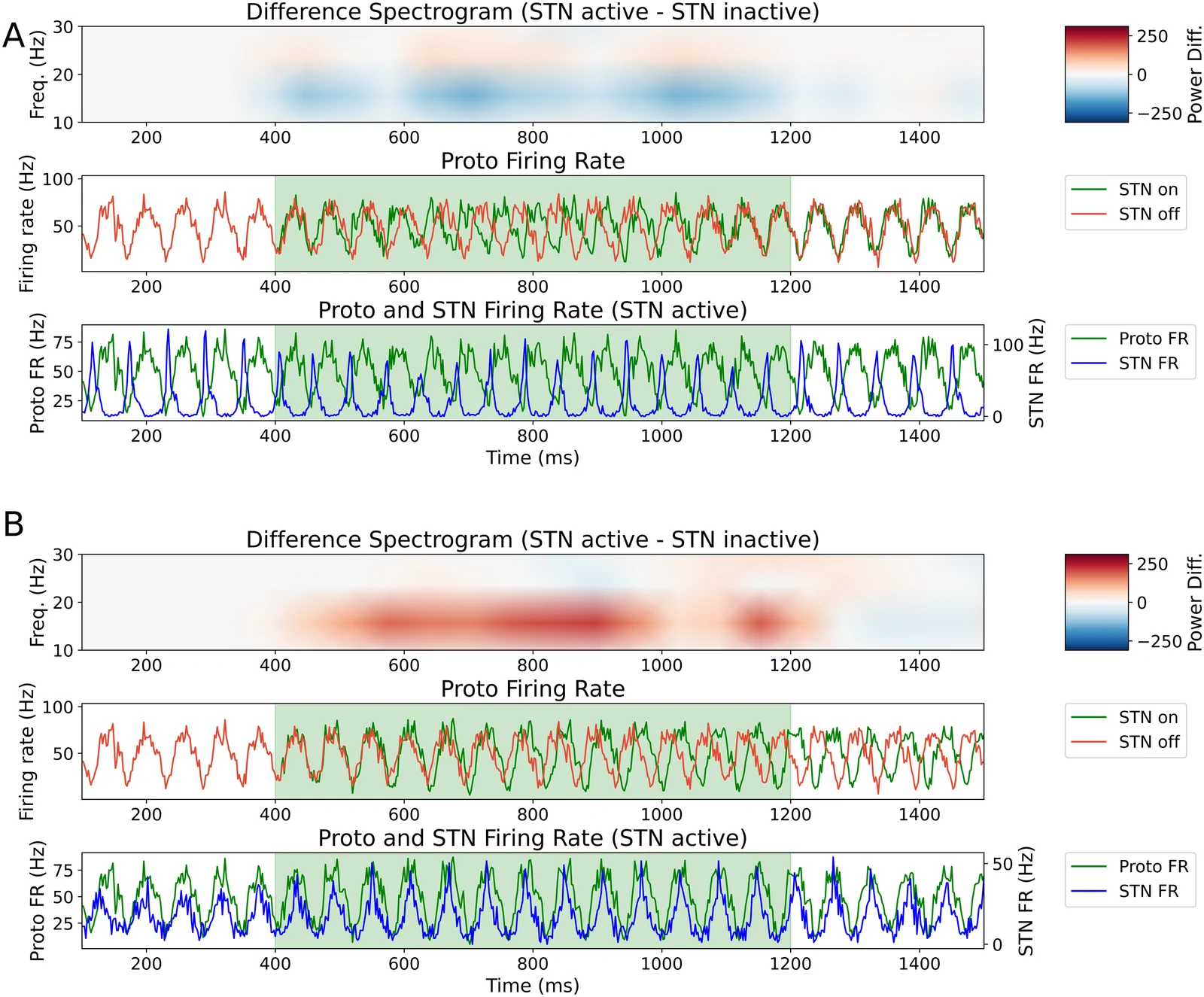
Effects of activation of STN→Proto connections on beta oscillations. (A) LIF STN configuration: turning on the STN→Proto connection (green region) decreases beta amplitude and establishes an anti-phase relationship between STN and Proto firing rates. (B) QIF STN configuration: the same intervention enhances beta amplitude and produces a different phase locking of STN and Proto.

To understand the mechanistic basis of how different STN neuron models affect beta oscillations, we performed activation experiments in the spiking networks. Fig 12 shows the effects of turning on the STN-to-Proto connection (green shaded region) for both LIF and QIF STN models. In the case of the LIF STN network (Fig 12A), activating the STN→Proto connection leads to a decrease in beta oscillation amplitude, as evidenced by both the blue shading in the difference spectrogram (top panel) and the comparison of the time series of Proto firing rates with (green) and without (orange) STN activation (middle panel). The bottom panel reveals that the phase difference between STN and Proto populations is 142.6 degrees during activation of the STN-to-Proto connection. In contrast, the QIF STN network (Fig 12B) shows the opposite effect: turning on the STN→Proto connection enhances beta oscillation amplitude and also affects the oscillation frequency slightly. The phase difference analysis (bottom panel) demonstrates that QIF STN establishes a markedly different phase relationship with Proto, with a phase difference of only 11.3 degrees. These results corroborate the idea that single neuron dynamics of STN determine the phase coupling with Proto, which in turn controls whether the subthalamopallidal loop enhances or suppresses beta oscillations generated by the pallidostriatal circuit.

**Fig 13:**
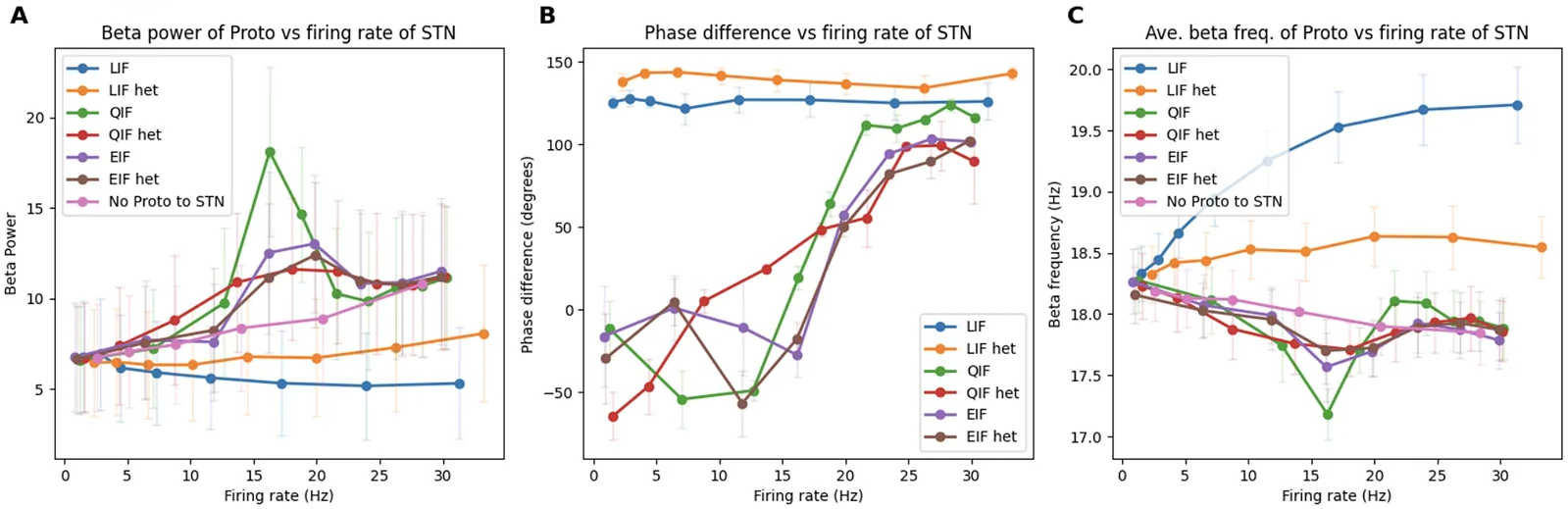
Effects of STN firing rate on beta power and phase. (A) Beta power in Proto versus STN firing rate for different network configurations. The baseline case without Proto→STN feedback (pink) shows a gradual increase in beta power with STN firing rate. With Proto→STN coupling, LIF STN neurons (blue) suppress beta power below baseline, while QIF (green) and EIF (purple) STN neurons enhance it, with a peak at at intermediate firing rates. Heterogeneous input conditions (LIF het, QIF het, EIF het) dampen the respective effects. (B) Phase difference between Proto and STN populations versus STN firing rate. LIF and LIF het neurons maintain a consistently large phase difference ($\sim 125-145^{\circ }$), while QIF and EIF neurons exhibit near-zero or negative phase differences at low firing rates, transitioning to larger values at higher rates. Error bars represent standard error across multiple seeds. (C) Average beta frequency of Proto versus STN firing rate. Beta frequency remains similar across all network configurations (17–20 Hz).

To systematically examine the relationship between STN firing rate, STN-Proto phase locking, and beta power modulation, we conducted parameter sweeps by varying the external input current to STN neurons across multiple network configurations. Fig 13 presents the results of this comprehensive analysis. Panel A shows how beta power in Proto varies with STN firing rate across various settings. The baseline condition without Proto→STN feedback (pink) shows a gradual increase in beta power with STN firing rate due a direct increase in the excitatory drive onto Proto. When Proto→STN feedback is present, the relationship becomes model-dependent: LIF STN neurons suppress beta power below baseline across all firing rates, while QIF and EIF STN neurons enhance it, particularly over an intermediate range of firing rates. Introducing heterogeneity in STN input currents (LIF het, QIF het, EIF het) partially dampens these effects in each case.

Fig 13B reveals the mechanistic basis for these differences. LIF neurons with and without heterogeneity maintain a consistently large phase difference with Proto ($\sim 125-145^{\circ }$) across all firing rates, while QIF and EIF neurons exhibit near-zero or negative phase differences at low firing rates. In the homogeneous QIF and EIF cases, the phase difference rises sharply at higher firing rates, corresponding to the weakening of the beta enhancement at high STN firing rates seen in Fig 13A. Heterogeneity smooths this transition, yielding a milder but broader enhancement of beta power. Fig 13C shows that the average beta frequency of Proto remains relatively stable across all STN firing rates and network configurations (17–20 Hz).

## Discussion

Our investigation into how neuronal model choice affects beta oscillation dynamics in the basal ganglia has revealed a fundamental mechanistic principle that reconciles seemingly contradictory findings in the Parkinson’s disease literature. Through systematic analysis spanning rate models, single neuron analysis, and spiking network simulations, we demonstrated that the phase relationship between STN and Proto populations serves as the critical determinant of whether the subthalamopallidal loop enhances or suppresses beta oscillations originating in the pallidostriatal circuit. Quadratic integrate-and-fire (QIF) STN neurons demonstrate stable in-phase relationships with inhibitory input, while leaky integrate-and-fire (LIF) STN neurons exhibit anti-phase locking. These neuron-level distinctions translate to population-level dynamics, determining whether the subthalamopallidal loop acts as a beta amplifier or suppressor.

Our rate model and spiking network simulations provided converging evidence that phase relationships directly control oscillation amplitude, with experiments demonstrating that identical circuit architectures produce opposite effects on beta power depending solely on STN neuron model choice. Parameter sweeps confirmed that these phase preferences remain consistent across physiologically relevant firing rate ranges, even when neurons are not strictly phase-locked to network rhythms.

The finding that QIF STN neurons establish in-phase relationships with Proto while LIF STN neurons prefer anti-phase coupling provides a direct mechanistic explanation for the divergent results between [39] and [40]. Rather than representing fundamental disagreements about circuit mechanisms, these studies’ contradictory conclusions likely stemmed from different neuronal model implementations. This observation underscores the point that careful consideration of neuronal model selection is crucial when investigating oscillatory phenomena in neural circuits.

Because STN neurons excite their Proto counterparts, an in-phase relationship in their firing tends to strengthen ongoing Proto oscillations, and this effect manifests with QIF STN neurons. Alternatively, with anti-phase locking, STN excitation arrives at troughs in Proto firing, diminishing these minima, and wanes at peaks in Proto firing, removing any potential boosting effect. Thus, anti-phase STN-Proto relationships lead to suppression of Proto oscillations, as we have seen with LIF STN neurons. In the LIF case, input heterogeneity dampens oscillations in STN firing, thereby weakening the impact of anti-phase STN firing on Proto beta (Fig 10).

The link between a particular STN model $v^{\prime }=I+f(v)-I_{inh}$ and the STN-Proto phase relationship that it produces derives from the shape of the curve *I* + *f*(*v*)=0. This curve determines where and when *dv*/*dt* slows down and how this deceleration is affected by inhibition, which combine to set the range of input phases over which a spike can occur (Fig 6). We can expect that qualitatively similar ideas will arise in higher-dimensional neuronal models, although we decided to exclude these from this study both to stay true to the original works on this topic [39,40] and to avoid drowning in degrees of freedom and parameter choices that could obscure the fundamental effects that we have described.

The time-to-spike (TTS) framework developed here represents a significant extension of previous applications [45,46], demonstrating that TTS can be redefined as a map based on comparison with a reference signal instead of based directly on time elapsed since the last spike as was done previously. By redefining TTS based on temporal offset from periodic inhibition, we captured phase dynamics while reducing complex continuous dynamics to mathematically tractable discrete maps. This approach is particularly well-suited for excitatory-inhibitory networks in which sequential interactions provide natural discrete events for mapping. In many other studies, phase response curves (PRCs) have been used to analyze related questions about phase locking in coupled or driven systems. We did not pursue an analysis based on PRCs because the coupling in our model networks is not weak. The strong coupling framework lacks a mathematical analogue to the PRCs used for weak coupling, and in our case the TTS map fills that gap.

An important consideration emerging from our literature review is the potential for species-specific differences in basal ganglia dynamics. Experiments supporting STN’s role in beta generation [17,18] were conducted in primates, while studies showing limited STN effects on beta [27] and supporting pallidostriatal origins of beta oscillations [5,28–34] used non-primates. This pattern suggests that there may be differences in STN neuron excitability between species, which could explain apparent contradictions through the phase relationship mechanisms we identified. Direct measurement of phase relationships between STN and Proto populations during beta oscillations in both primate and rodent models could be used to test for the presence of the relationships that we predict. Additionally, applying periodic inhibitory stimulation to single STN neurons in slice preparations could directly test whether primate and rodent STN neurons exhibit QIF-like and LIF-like dynamics, respectively, at least with regard to their phase-locking properties. Testing periodic inhibition at dendritic versus somatic locations could reveal whether dendritic integration, potentially more extensive in primate STN neurons, contributes to QIF-like behavior.

While computational efficiency often drives the use of simplified integrate-and-fire models, our findings demonstrate that this choice fundamentally alters biological conclusions. The field would benefit from systematic comparisons between simplified and conductance-based models, specifically examining whether they preserve critical dynamical properties like phase relationships. The demonstration that single neuron dynamics can fundamentally alter population-level pathological activity represents a broader principle that should be considered from both theoretical and experimental perspectives. On the computational side, seemingly minor modeling choices can have profound consequences for model network behavior and clinical interpretation [6]. This insight underscores the importance of validating computational predictions against multiple experimental measures and considering how model assumptions might influence conclusions about disease mechanisms and therapeutic targets. On the biological side, these results offer a reminder that even within large networks of coupled neurons, the properties of individual neurons can influence emergent dynamics and remain important to elucidate.

Beyond Parkinson’s disease, our work provides insights into how pathological oscillations may emerge and be modulated in other neurological and psychiatric conditions. The principle that phase relationships between brain regions critically determine oscillatory power could apply to epilepsy, schizophrenia, and other disorders characterized by abnormal neural synchronization [47–50].

## Supporting information

S1 Appendix Appendix A: Proof that the LIF STN model fires during the inhibition-off period. Appendix B: Proof that the QIF STN model fires during the inhibition-on period. Appendix C: Parameter sweep of steady-state phase relationships. Appendix D: Supporting figures for TTS. Appendix E: Single neuron dynamics under sinusoidal inhibition. (PDF)

S1 FigSteady-state phase relationships between STN and Proto populations as a function of excitatory drive *I* and inhibition amplitude *a.*(A) LIF neurons with *V*_reset_ = 0 exhibit phase relationships ranging from $90^{\circ }$ (anti-phase, blue) to $270^{\circ }$ (predominantly anti-phase, yellow). (B) QIF neurons display phase values from $-90^{\circ }$ to $130^{\circ }$, showing stronger tendency toward in-phase relationships. Red lines show theoretical boundaries where phase-locking ceases to exist, derived from existence conditions (see Appendices A and B). Between these boundaries, neurons maintain stable phase-locking to periodic inhibition, with the steady-state phase varying smoothly with parameters (indicated by color). The close agreement between analytical predictions (red lines) and numerically observed boundaries validates the time-to-spike framework. For LIF neurons, the boundaries are *V*(*T*; *T*/4) = *V*_th_ (solid) and *V*(*T*; 3*T*/4) = *V*_th_ (dashed). For QIF neurons, the boundaries are *V*(*T*; *T*/4) = *V*_th_ (dashed) and $V(T;-T/4)=V_{\text{th}}$ (solid). Parameters: *T* = 2, *V*_th_ = 1.0, $V_{r}=0$ (LIF), $V_{r}=-0.2$ (QIF).(TIF)

S2 FigReturn maps of the time-to-spike (TTS) function for LIF and QIF neuron models.Each panel plots $t_{n+1}^{in}=T+t_{n}^{in}-TTS(t_{n}^{in})$ against $t_{n}^{in}$. The dashed grey diagonal marks fixed points; intersections with the colored curve correspond to phase-locked solutions. (A) LIF neuron (blue): the stable fixed point lies in the inhibition-off region, consistent with Fig 7B. (B) QIF neuron (red): the stable fixed point lies in the inhibition-on region, consistent with Fig 7D. In both panels, a slope between 0 and 1 at the intersection confirms stability, in agreement with the contraction and dilation regions shown in Fig 7E. Parameters: *T* = 2, $V_{th}=1$, $V_{r}=0$ (LIF), $V_{r}=-0.2$ (QIF).(TIF)

S3 FigSteady-state phase as a function of input current *I* and oscillation period *T* for LIF, QIF, and EIF neuron models.Color indicates the phase (in degrees) of the stable fixed point of the TTS map, with the colormap centered at $90^{\circ }$ to highlight the boundary between in-phase (blue, $<90^{\circ }$) and anti-phase (red, $>90^{\circ }$) regimes. Gray regions denote parameter combinations where no stable phase-locked solution exists. (A) LIF neurons (*a* = 0.5): phase values remain in the anti-phase range across all *I*–*T* combinations explored. (B) QIF neurons (*a* = 0.01) and (C) EIF neurons (*a* = 0.06): phase values are predominantly in-phase (blue) at shorter periods, with a gradual shift toward anti-phase values as *T* increases. Parameters as in Table 3.(TIF)

S4 FigLIF and QIF neuron dynamics and TTS functions under sinusoidal inhibitory forcing.Inhibition follows $I_{inh}(t)=a(\sin(2\pi t/T+\pi /2)+1)$, peaking at *t* = 0. All other parameters match Fig 7: *T* = 2, $V_{th}=1$, $V_{r}=0$ (LIF), $V_{r}=-0.2$ (QIF). (A) Membrane potential of an LIF neuron (green) overlaid with the inhibition waveform (dashed). Spikes occur preferentially during the inhibition-off phase, consistent with the anti-phase preference seen under square-wave forcing (Fig 6C). (B) TTS function for the LIF neuron as a function of phase offset. The stable fixed point (intersection with the red dashed line *T* = 2) lies in the inhibition-off region (right of the green dashed line), confirming anti-phase locking. (C) Membrane potential of a QIF neuron (blue) overlaid with the inhibition waveform. Spikes occur preferentially during the inhibition-on phase, consistent with the in-phase preference seen under square-wave forcing (Fig 6A). (D) TTS function for the QIF neuron. The stable fixed point lies in the inhibition-on region (left of the green dashed line), confirming in-phase locking.(TIF)

S5 FigSteady-state phase relationships under sinusoidal inhibition as a function of excitatory drive *I* and inhibition amplitude *a* for LIF, QIF, and EIF neuron models.Inhibition follows $I_{inh}(t)=a(\sin(2\pi t/T+\pi /2)+1)$. Color indicates the phase (in degrees) of the stable fixed point of the TTS map, with the colormap centered at $90^{\circ }$ to highlight the boundary between in-phase (blue, $<90^{\circ }$) and anti-phase (red, $>90^{\circ }$) regimes. Gray regions denote parameter combinations where no stable phase-locked solution exists. Parameters for each model are given in Table 3. (A) LIF neurons: phase values remain in the anti-phase range ($\sim 90^{\circ },270^{\circ }$) across all parameter combinations explored, consistent with the square-wave results (Fig 9A). (B) QIF neurons and (C) EIF neurons: phase values are predominantly in-phase (blue), with anti-phase solutions appearing as inhibition amplitude *a* increases, consistent with the square-wave results (Fig 9C).(TIF)
