# Supplementary material for: Reconciling contradictory models of subthalamic nucleus contributions to basal ganglia beta oscillations
Source: PLoS Comput Biol. 2026 Aug 17;22(8):e1013942. doi: 10.1371/journal.pcbi.1013942 (PMC13541254; doi:10.1371/journal.pcbi.1013942)
Supplement: Supplementary file 1 [file pcbi.1013942.s001.pdf]

Supporting Information, S1 Appendix

Appendix A – Proof that the LIF STN model fires during the inhibition-off period. We study the existence and stability of fixed points of the time-to-spike map arising in the inhibition-on and -off periods for an LIF neuron driven with periodic inhibition. For convenience, we rescale time to units of the membrane time constant and we first obtain the solution of the LIF equation for a general input $\tilde{E}$. The equation is given by $V' = \tilde{E} - V$, and with data $V(t_0) = V_0$ the solution is

$$V(t) = \tilde{E}(1 - e^{t_0 - t}) + V_0 e^{t_0 - t}. \quad \text{(A1)}$$

Let I and a be the input current and inhibition strength, respectively. Then $\tilde{E} = I$ when inhibition is off and $\tilde{E} = I - a$ when inhibition is on. Suppose that when V reaches the threshold at $V = V_{th} = 1$ it is reset to $V = 0$ and that we start the solution with $V(0) = 0$, with the neuron having just been reset after a spike.

We will proceed by showing that the TTS map acts as a contraction everywhere on the interval of initial conditions corresponding to spiking and being reset with inhibition off, $t^{in} \in (T/4, 3T/4)$; note that initially, we suppress the subscript n of t_n^{in} since we consider a single iteration of the map. This result will imply that any fixed point arising in the time range corresponding to inhibition off will be stable. Recall from the section *Single neuron dynamics and phase relationships under periodic inhibition*, as visualized by Fig 5, that t^{in} is defined as the time difference between the spike of the excitatory neuron and the mid-point of the next time interval when its inhibitory input is on. In this case, the first positive time when inhibition turns on will be at $t = t^{in} - T/4$. The next inhibition-off time will be $t^{in} - T/4 + T/2 = t^{in} + T/4$ and inhibition will remain off up to $t^{in} + 3T/4$. The full time domain of interest in this case is $[0, t^{in} + 3T/4]$, because we want the neuron fire again during this inhibition-off period. Thus, the relevant piecewise system takes the form

$$V' = \begin{cases} I - a - V & \text{if} \quad t^{in} - \frac{T}{4} < t < t^{in} + \frac{T}{4}, \\ I - V & \text{otherwise.} \end{cases}$$

Consider the moments when inhibition turns on ($t = t^{in} - \frac{T}{4}$) and off ($t = t^{in} + \frac{T}{4}$), respectively. The solution formula (A1) gives us

$$\begin{aligned} V(t^{in} - \frac{T}{4}) &= I(1 - e^{-t^{in} + \frac{T}{4}}), \\ V(t^{in} + \frac{T}{4}) &= (I - a)(1 - e^{-\frac{T}{2}}) + V(t^{in} - \frac{T}{4})e^{-\frac{T}{2}} \\ &= I - a(1 - e^{-\frac{T}{2}}) - Ie^{-t^{in} - \frac{T}{4}} \end{aligned}$$

Since we are assuming that firing occurs in the inhibition-off period, the voltage reaches the firing threshold after time $t^{in} + \frac{T}{4}$, say at $t = t^{in} + \frac{T}{4} + \tilde{t}^f(t^{in})$. Applying equation (A1) at the moment the neuron spikes with $t_0 = t^{in} + \frac{T}{4}$ and grouping the

exponential terms gives us

$$\begin{aligned}
1 &= I + (V(t^{in} + \frac{T}{4}) - I)e^{-\tilde{t}^f} \text{ and} \\
\tilde{t}^f(t^{in}) &= \ln\left(\frac{I - V(t^{in} + \frac{T}{4})}{I-1}\right) \\
&= \ln\left(\frac{I - (I - a(1-e^{-\frac{T}{2}}) - Ie^{-t^{in}-\frac{T}{4}})}{I-1}\right) \\
&= \ln\left(\frac{a(1-e^{-\frac{T}{2}}) + Ie^{-t^{in}-\frac{T}{4}}}{I-1}\right).
\end{aligned}$$

Now, suppose that $t_n^{in,1} < t_n^{in,2}$ are two possible firing times during the inhibition-off period. Since $TTS(t_n^{in,i}) = t_n^{in,i} + \frac{T}{4} + \tilde{t}^f(t_n^{in,i})$,

$$\begin{aligned}
TTS(t_n^{in,2}) - TTS(t_n^{in,1}) &= t_n^{in,2} - t_n^{in,1} + \ln\left(\frac{a(1-e^{-\frac{T}{2}}) + Ie^{-t_n^{in,2}-\frac{T}{4}}}{a(1-e^{-\frac{T}{2}}) + Ie^{-t_n^{in,1}-\frac{T}{4}}}\right) \\
&= \ln\left(\frac{e^{t_n^{in,2}}}{e^{t_n^{in,1}}}\frac{a(1-e^{-\frac{T}{2}}) + Ie^{-t_n^{in,2}-\frac{T}{4}}}{a(1-e^{-\frac{T}{2}}) + Ie^{-t_n^{in,1}-\frac{T}{4}}}\right) \\
&= \ln\left(\frac{a(1-e^{-\frac{T}{2}})e^{t_n^{in,2}} + Ie^{-\frac{T}{4}}}{a(1-e^{-\frac{T}{2}})e^{t_n^{in,1}} + Ie^{-\frac{T}{4}}}\right) > 0
\end{aligned}$$

and, using the time-to-spike map definition (6),

$$\begin{aligned}
0 < t_{n+1}^{in,2} - t_{n+1}^{in,1} &= t_n^{in,2} - t_n^{in,1} - (TTS(t_n^{in,2}) - TTS(t_n^{in,1})) \\
&< t_n^{in,2} - t_n^{in,1}.
\end{aligned}$$

Thus, we have shown that the map is a contraction on the inhibition-off period, and hence any fixed point within that period is stable.

For such a fixed point to exist, we need

$$\begin{aligned}
t_{n+1}^{in} &= t_n^{in} + T - TTS(t_n^{in}) \\
&= t_n^{in} + T - (t_n^{in} + \frac{T}{4} + \tilde{t}_n^f) \\
&= \frac{3}{4}T - \ln\left(\frac{a(1-e^{-\frac{T}{2}}) + Ie^{-t_n^{in}-\frac{T}{4}}}{I-1}\right).
\end{aligned}$$

Thus, the fixed point equation becomes

$$\begin{aligned}
t_{ss}^{in} &= \frac{3}{4}T - \ln\left(\frac{a(1-e^{-\frac{T}{2}}) + Ie^{-t_{ss}^{in}-\frac{T}{4}}}{I-1}\right) \\
&= \frac{3}{4}T - \ln\left(\frac{e^{-t_{ss}^{in}}(a(1-e^{-\frac{T}{2}})e^{t_{ss}^{in}} + Ie^{-\frac{T}{4}})}{I-1}\right) \\
&= \frac{3}{4}T - \left(-t_{ss}^{in} + \ln\frac{a(1-e^{-\frac{T}{2}})e^{t_{ss}^{in}} + Ie^{-\frac{T}{4}}}{I-1}\right),
\end{aligned}$$

from which we derive

$$\frac{a(1-e^{-\frac{T}{2}})e^{t_{ss}^{in}} + Ie^{-\frac{T}{4}}}{I-1} = e^{\frac{3}{4}T}$$

and hence

$$t_{ss}^{in} = \ln\left(\frac{I(e^{\frac{3}{4}T} - e^{-\frac{T}{4}}) - e^{\frac{3}{4}T}}{a(1 - e^{-\frac{T}{2}})}\right). \tag{A2}$$

So, the fixed point exists, and is an increasing function of I and a decreasing function of a, if this expression is well-defined and lies in the interval $(T/4, 3T/4)$, corresponding to the inhibition-off period. We are only interested in the case when $I > 1$, so that the neuron can fire, and the conditions $t_{ss}^{in} \in (T/4, 3T/4)$ are equivalent to the conditions

$$I(1 - e^{-T}) - a(1 - e^{-T/2}) < 1 < I(1 - e^{-T}) - a(1 - e^{-T/2})e^{-T/2}. \tag{A3}$$

Thus, the LIF model gives a stable fixed point with inhibition-off firing when conditions (A3) hold or, rewritten in terms of conditions on I or a,

$$\begin{aligned} \frac{1 + a(1 - e^{-T/2})e^{-T/2}}{1 - e^{-T}} < \quad I \quad &< \frac{1 + a(1 - e^{-T/2})}{1 - e^{-T}}, \text{ or} \\ \frac{I(1 - e^{-T}) - 1}{1 - e^{-T/2}} < \quad a \quad &< \frac{(I(1 - e^{-T}) - 1)e^{T/2}}{1 - e^{-T/2}}. \end{aligned}$$

To complete our argument, we want to show that the map acts locally as a dilation on the region of initial conditions with inhibition on. This result will imply that any fixed point in the inhibition-on region is unstable. To proceed, assume that the neuron spiked with inhibition on. Then, the next inhibition-off time would be $t^{in} + \frac{1}{4}T$, and the next inhbition on time would be $t^{in} + \frac{3}{4}T$; thus,

$$V' = \begin{cases} I - V & \text{if} \quad t^{in} + \frac{1}{4}T < t < t^{in} + \frac{3}{4}T, \\ I - a - V & \text{otherwise.} \end{cases}$$

Following the steps in the last case, we have:

$$\begin{aligned} V(t^{in} + \frac{1}{4}T) &= (I - a)(1 - e^{-t^{in} - \frac{1}{4}T}) \\ V(t^{in} + \frac{3}{4}T) &= I(1 - e^{-\frac{1}{2}T}) + (I - a)(1 - e^{-t^{in} - \frac{1}{4}T})e^{-\frac{1}{2}T} \\ &= I - ae^{-\frac{1}{2}T} - (I - a)e^{-t^{in} - \frac{3}{4}T} \end{aligned}$$

and

$$\begin{aligned} 1 &= (I - a) + (V(t^{in} + \frac{3}{4}T) - (I - a))e^{-\tilde{t}^f} \\ &= (I - a) + (a(1 - e^{-\frac{1}{2}T}) - (I - a)e^{-t^{in} - \frac{3}{4}T})e^{-\tilde{t}^f} \text{ such that} \\ \tilde{t}^f &= \ln\left(\frac{-a(1 - e^{-\frac{1}{2}T}) + (I - a)e^{-t^{in} - \frac{3}{4}T}}{I - a - 1}\right). \end{aligned}$$

Proceeding as in the previous case and using $t_n^{in,2} > t_n^{in,1}$, we have

$$t_{n+1}^{in,2} - t_{n+1}^{in,1} = t_n^{in,2} - t_n^{in,1} - \ln\left(\frac{-a(1 - e^{-\frac{1}{2}T})e^{t^{in,2} + \frac{3}{4}T} + (I - a)}{-a(1 - e^{-\frac{1}{2}T})e^{t^{in,1} + \frac{3}{4}T} + (I - a)}\right) > t_n^{in,2} - t_n^{in,1}.$$

Hence, the map is a dilation on initial conditions in the inhibition-on region and cannot have a stable fixed point there.

Appendix B – Proof that the QIF STN model fires during the inhibition-on period. The goal of this section is to show that for the QIF model, there exists a fixed point of the time-to-spike map inside the inhibition-on interval and to derive conditions under which this fixed point is stable. Similar to what we did in the LIF case, we rescale time to units of the membrane time constant and compute the solution of the QIF equation with an arbitrary input $\tilde{E}$.

Case 1: The nullcine stays above the x-axis. We first consider the case $I > 0$, $a > 0$, and $I - a > 0$, so that the V-nullcline does not intersect the axis when inhibition is on. If we ignore the threshold and associated reset, for $\tilde{E} > 0$, we have:

$$V' = V^2 + \tilde{E}, \quad V(t_0) = V_0$$

with solution

$$V(t) = \sqrt{\tilde{E}} \tan\left(\sqrt{\tilde{E}}(t - t_0) + \tan^{-1} \frac{V_0}{\sqrt{\tilde{E}}}\right), \tag{A4}$$

which we can invert to obtain

$$t = \frac{1}{\sqrt{\tilde{E}}} \left(\tan^{-1} \frac{V(t)}{\sqrt{\tilde{E}}} - \tan^{-1} \frac{V_0}{\sqrt{\tilde{E}}}\right) + t_0. \tag{A5}$$

Again, $\tilde{E} = I$ when inhibition is off and $\tilde{E} = I - a$ when inhibition is on. Suppose that the model neuron fires with inhibition on. The voltage ODE becomes

$$V' = \begin{cases} I + V^2 & \text{if } t^{in} + \frac{T}{4} < t < t^{in} + \frac{3T}{4} \\ I - a + V^2 & \text{otherwise} \end{cases}$$

where $t^{in} \in (-T/4, T/4)$ is positive if the firing happens before the midpoint of the inhibition-on interval and negative if it happens afterwards.

Consider the voltages the moments when inihibition turns off ($t = t^{in} + \frac{T}{4}$) and on ($t = t^{in} + \frac{3T}{4}$), respectively. The solution formula (A4) gives

$$\begin{aligned} V\left(t^{in} + \frac{T}{4}\right) &= \sqrt{I-a} \tan\left(\sqrt{I-a}\left(t^{in} + \frac{T}{4}\right) + \tan^{-1} \frac{V_r}{\sqrt{I-a}}\right) \\ &:= \sqrt{I-a} \tan A_1, \end{aligned} \tag{A6}$$

$$\begin{aligned} V\left(t^{in} + \frac{3T}{4}\right) &= \sqrt{I} \tan\left(\sqrt{I} \cdot \frac{T}{2} + \tan^{-1} \frac{V\left(t^{in} + \frac{T}{4}\right)}{\sqrt{I}}\right) \\ &:= \sqrt{I} \tan A_2, \end{aligned} \tag{A7}$$

where A_1 and A_2 will be used for the stability argument below.

Existence: Consider t^{in} as a parameter, the solution formula (A4) applied from the time $t = t^{in} + 3T/4$ when inhibition turns back on up to time T gives

$$V(T; t^{in}) = \sqrt{I-a} \tan\left(\sqrt{I-a} \cdot \left(\frac{T}{4} - t^{in}\right) + \tan^{-1} \frac{V(t^{in} + \frac{3T}{4})}{\sqrt{I-a}}\right).$$

To establish the existence of a fixed point, we want to show that the trajectory with $t^{in} = \frac{T}{4}$ does not reach the firing threshold by the time inhibition turns on again at $t = T$, while the trajectory with $t^{in} = -\frac{T}{4}$, ends up past the firing threshold at $t = T$ (ignoring reset). In other words, we want

$$\begin{aligned} &V(T; T/4) \\ =&\sqrt{I} \tan\left[\sqrt{I}T/2 + \tan^{-1} \frac{\sqrt{I-a} \tan(\sqrt{I-a}T/2 + \tan^{-1}(V_r/\sqrt{I-a}))}{\sqrt{I}}\right] < V_{th} \end{aligned} \tag{A8}$$

and

$$
\begin{aligned}
&V(T;-T/4)\\
=&\sqrt{I-a}\tan\left[\sqrt{I-a}T/2+\tan^{-1}\frac{\sqrt{I}\tan(\sqrt{I}T/2+\tan^{-1}(V_r/\sqrt{I}))}{\sqrt{I-a}}\right]>V_{th}.
\end{aligned}
\tag{A9}
$$

If conditions (A8) and (A9) are satisfied, then by the intermediate value theorem, there exists $t_{ss}^{in}\in\left(-\frac{T}{4},\frac{T}{4}\right)$ such that

$$V(T;t_{ss}^{in})=V_{th},$$

with reset back to $V_r=V(0;t_{ss}^{in})$, so the corresponding solution is phase-locked to the periodic inhibition with firing during the inhibition-on period; that is, this solution is our desired fixed point.

Intuitively, we expect it to be easy to satisfy both (A8) and (A9): If the inhibition-on period comes first, as for (A8), then the trajectory cannot advance much in V as long as $I-a$ is relatively small (i.e., due to the ghost of the saddle node bifurcation at $I-a=0$), and then it has a long way to go to reach threshold once inhibition is off. On the other hand, if inhibition-off comes first, as for (A9), then the trajectory can freely advance past what would have been a bottleneck had inhibition been present, and then it is relatively free to progress to threshold once inhibition comes on.

To demonstrate this intuition more analytically, let us first consider the solution that has $t^{in}=T/4$, which starts from $t=0$ with $V(0)=V_r<0$. To simplify notation, set $V_{th}=1$. After time T, this solution's voltage value is given by the left hand side of inequality (A8). Suppose we approximate $V(T/2)$, when inhibition turns off, by 0. Then $V(T)\approx\sqrt{I}\tan(\sqrt{I}T/2)$. Next, consider the solution $\tilde{V}(t)$ that has $t^{in}=-T/4$, with $\tilde{V}(0)=V_r<0$. This solution has $\tilde{V}(T/2)=\sqrt{I}\tan(\sqrt{I}T/2+\tan^{-1}(V_r/\sqrt{I}))$. To find $\tilde{V}(T)$, we integrate $\hat{V}'=I-a+(\hat{V})^2$ with $\hat{V}(0)=\tilde{V}(T/2)$, up to time $T/2$. Assuming that $I-a$ is small, we approximate $\tilde{V}(T)=\hat{V}(T/2)\approx\dfrac{\tilde{V}(T/2)}{1-(T/2)\tilde{V}(T/2)}$, from the solution of $\hat{V}'=(\hat{V})^2$.

Thus, we ask, under what conditions will we have $V(T)<1$, so that (A8) holds, and $\tilde{V}(T)>1$, so that (A9) holds? Using our approximation, the latter condition is equivalent to $\tilde{V}(T/2)>1/(1+T/2)$. Note that $\tilde{V}(T/2)<V(T)$, with $\tilde{V}(T/2)\uparrow V(T)$ as $V_r\uparrow 0$. Thus, since $1>1/(1+T/2)$, for any fixed T, we can always satisfy these conditions by choosing $\sqrt{I}\tan(\sqrt{I}T/2)\in(1/(1+T/2),1)$ and V_r not too far below 0.

Stability: We show that any fixed point in the inhibition-on region is stable by calculating the derivative of the map TTS. Equation (A5) with $\tilde{E}=I-a$, $V_0=V\left(t^{in}+\frac{3T}{4}\right)$, $t=TTS(t^{in})$, $t_0=t^{in}+\frac{3T}{4}$, and $V(TTS(t^{in}))=V_{th}$ yields

$$TTS(t^{in})=\frac{1}{\sqrt{I-a}}\left(\tan^{-1}\frac{V_{th}}{\sqrt{I-a}}-\tan^{-1}\frac{V\left(t^{in}+\frac{3T}{4}\right)}{\sqrt{I-a}}\right)+\left(t^{in}+\frac{3T}{4}\right).$$

We apply chain rule to compute the derivative of TTS:

$$
\begin{aligned}
\frac{dA_1}{dt^{in}}&=\frac{d}{dt^{in}}\left(\sqrt{I-a}\left(t^{in}+\frac{T}{4}\right)+\tan^{-1}\frac{V_r}{\sqrt{I-a}}\right);\\
&=\sqrt{I-a}
\end{aligned}
$$

$$
\begin{aligned}
\frac{dV\left(t^{in}+\frac{T}{4}\right)}{dt^{in}}&=\frac{d}{dt^{in}}\left(\sqrt{I-a}\tan A_1\right)\\
&=\sqrt{I-a}\cdot\sec^2 A_1\cdot\sqrt{I-a}=(I-a)\sec^2 A_1;
\end{aligned}
$$

$$\begin{aligned}\frac{dA_2}{dt^{in}} &= \frac{d}{dt^{in}}\left(\sqrt{I}\cdot\frac{T}{2}+\tan^{-1}\frac{V\left(t^{in}+\frac{T}{4}\right)}{\sqrt{I}}\right)\\ &= \frac{1}{1+\left(\frac{V\left(t^{in}+\frac{T}{4}\right)}{\sqrt{I}}\right)^2}\cdot\frac{d}{dt^{in}}\left(\frac{V\left(t^{in}+\frac{T}{4}\right)}{\sqrt{I}}\right)\\ &= \frac{I}{I+(I-a)\tan^2 A_1}\cdot\frac{1}{\sqrt{I}}\cdot(I-a)\sec^2 A_1\\ &= \frac{\sqrt{I}(I-a)}{I-a\sin^2 A_1};\end{aligned}$$

$$\begin{aligned}\frac{dV\left(t^{in}+\frac{3T}{4}\right)}{dt^{in}} &= \frac{d}{dt^{in}}\left(\sqrt{I}\tan A_2\right)\\ &= \sqrt{I}\sec^2 A_2\cdot\frac{\sqrt{I}(I-a)}{I-a\sin^2 A_1}\\ &= \frac{I(I-a)\sec^2 A_2}{I-a\sin^2 A_1};\end{aligned}$$

$$\begin{aligned}\frac{d}{dt^{in}}TTS(t^{in}) &= \frac{1}{\sqrt{I-a}}\left(-\frac{1}{1+\left(\frac{\sqrt{I}\tan A_2}{\sqrt{I-a}}\right)^2}\cdot\frac{d}{dt^{in}}\frac{\sqrt{I}\tan A_2}{\sqrt{I-a}}\right)+1\\ &= 1-\frac{I(I-a)\sec^2(A_2)}{(I-a+I\tan^2 A_2)(I-a\sin^2 A+1)}\\ &= 1-\frac{I^2-aI}{I^2-aI(\cos^2 A_2+\sin^2 A_1)+a^2\cos^2 A_2\sin^2 A_1}\\ &> 1-\frac{I^2-aI}{I^2-aI(\cos^2 A_2+\sin^2 A_1)}\overset{(*)}{>}1-\frac{I^2-aI}{I^2-aI}=0.\end{aligned}$$

Inequality (*) would be true if the following conditions are satisfied:

1. $A_1, A_2 > 0$
2. $\frac{V(t^{in}+\frac{T}{4})}{\sqrt{I-a}} < \frac{V(t^{in}+\frac{3T}{4})}{\sqrt{I}}$

That is, under these conditions, by definitions (A6) and (A7), we have

$$\begin{aligned}\tan A_1 &< \tan A_2,\\ 0 < A_1 &< A_2 < \frac{\pi}{2}.\end{aligned}$$

Since $\cos^2(x)$ is a decreasing function in $[0, \frac{\pi}{2}]$, it follows that

$$\cos^2 A_2 + \sin^2 A_1 < \cos^2 A_1 + \sin^2 A_1 = 1,$$

which establishes inequality (*).

Note that $A_2 > 0$ from condition (a) is equivalent to $V(t^{in} + 3T/4) > 0$ by definition (A7). This is usually satisfied in practice, because when inhibition turns on, the voltage should be close enough to the firing threshold, and we always set the threshold to be

positive. Similarly, condition (b) means that the voltage during the inhibition-off period should increase sufficiently. The condition $A_1 > 0$ could be replaced by $A_2 + A_1 > 0$ with $A_1 \in (-\pi/2, 0]$. This condition certainly holds if V_r is not too far below 0.

Since $I > a$, the fractional term in $\frac{d}{dt^{in}} TTS(t^{in})$ is positive, which implies $0 < \frac{d}{dt^{in}} TTS(t^{in}) < 1$. Therefore, the fixed point of the map $t^{in}_{n+1} = t^{in}_n + T - TTS(t^{in}_n)$ during inhibition is stable under conditions (a) and (b), as the derivative of the map satisfies $|f'| < 1$.

Case 2: The nullcine intersects the axis We now assume $I > 0$, $a > 0$, and $I - a < 0$. As in the previous case, we have

$$V' = \begin{cases} I + V^2 & \text{if } t^{in} + \frac{T}{4} < t < t^{in} + \frac{3T}{4} \\ I - a + V^2 & \text{otherwise} \end{cases}$$

We will also assume here that $V_r < -\sqrt{a - I}$, such that $V' > 0$ immediately after any reset regardless of its timing. Note that if inhibition turns on, giving $-\tilde{E} = a - I$, with $t = t_0$, $V(t_0) = V_0$, and $|V_0| > \sqrt{a - I}$, then $|V(t)| > \sqrt{a - I}$ remains true as long as inhibition stays on.

The general solution to the QIF equation $V' = V^2 + \tilde{E}$ with constant input $\tilde{E}$ is given by equation (A4) when $\tilde{E} > 0$. When $\tilde{E} < 0$ and $|V_0| > \sqrt{-\tilde{E}}$, the solution is

$$V(t) = \sqrt{-\tilde{E}} \coth\left(\operatorname{arccoth} \frac{V_0}{\sqrt{-\tilde{E}}} - \sqrt{-\tilde{E}}\,(t - t_0) \right). \tag{A10}$$

We now use these solutions to compute the time-to-spike function. Using (A10) on the inhibition-on segment with $\tilde{E} = I - a < 0$, $V_0 = V_r$, $t_0 = 0$, and evaluating at $t = t^{in} + \frac{T}{4}$ gives:

$$\begin{aligned} V\left(t^{in} + \frac{T}{4}\right) &= \sqrt{a - I} \coth\left(\operatorname{arccoth} \frac{V_r}{\sqrt{a - I}} - \sqrt{a - I}\left(t^{in} + \frac{T}{4}\right)\right) \\ &:= \sqrt{a - I} \coth A_1. \end{aligned}$$

Using (A4) on the inhibition-off segment with $\tilde{E} = I > 0$ over a time duration $T/2$ starting from $t = t^{in} + T/4$ yields:

$$\begin{aligned} V\left(t^{in} + \frac{3T}{4}\right) &= \sqrt{I} \tan\left(\sqrt{I}\,\frac{T}{2} + \tan^{-1} \frac{V\left(t^{in} + \frac{T}{4}\right)}{\sqrt{I}} \right) \\ &:= \sqrt{I} \tan A_2. \end{aligned}$$

Existence: We will derive conditions for the existence of a fixed point under the assumption that $\sqrt{I} \tan A_2 > \sqrt{a - I}$. Considering t^{in} as a parameter, the solution formula (A10) applied from the time $t = t^{in} + 3T/4$ when inhibition turns back on up to time T thus gives

$$V(T; t^{in}) = \sqrt{a - I} \coth\left(\operatorname{arccoth} \frac{V(t^{in} + \frac{3T}{4})}{\sqrt{a - I}} - \sqrt{a - I} \cdot \left(\frac{T}{4} - t^{in} \right) \right).$$

To establish the existence of a fixed point, we want to show that the trajectory with $t^{in} = \frac{T}{4}$ does not reach the firing threshold by the time inhibition turns on again at $t = T$, while the trajectory with $t^{in} = -\frac{T}{4}$, ends up past the firing threshold at $t = T$

(ignoring reset). In other words, we want

$$\begin{aligned} &V(T;T/4)\\ =&\sqrt{I}\tan\left[\sqrt{I}T/2+\tan^{-1}\frac{\sqrt{a-I}\coth(\operatorname{arccoth}(V_r/\sqrt{a-I})-\sqrt{a-I}T/2)}{\sqrt{I}}\right]<V_{th} \end{aligned} \tag{A11}$$

and

$$\begin{aligned} &V(T;-T/4)\\ =&\sqrt{a-I}\coth\left[\operatorname{arccoth}\frac{\sqrt{I}\tan(\sqrt{I}T/2+\tan^{-1}(V_r/\sqrt{I}))}{\sqrt{a-I}}-\sqrt{a-I}T/2\right]>V_{th}. \end{aligned} \tag{A12}$$

Under our earlier assumption that $\sqrt{I}\tan A_2>\sqrt{a-I}$, if conditions (A11) and (A12) are satisfied, then by the intermediate value theorem, there exists $t_{ss}^{in}\in\left(-\frac{T}{4},\frac{T}{4}\right)$ such that

$$V(T;t_{ss}^{in})=V_{th},$$

with reset back to $V_r=V(0;t_{ss}^{in})$, so the corresponding solution is phase-locked to the periodic inhibition with firing during the inhibition-on period; that is, this solution is our desired fixed point.

Stablity: The time-to-spike function satisfies $V(\mathrm{TTS}(t^{in}))=V_{\mathrm{th}}$. Similarly to the previous case, we use (A10) with $t_0=t^{in}+3T/4, V_0=V(t^{in}+3T/4)$, and $\tilde{E}=I-a$ to calculate that when spiking occurs during the inhibition-on period,

$$\mathrm{TTS}(t^{in})=t^{in}+\frac{3T}{4}+\frac{1}{\sqrt{a-I}}\left[\operatorname{arccoth}\left(\frac{V\left(t^{in}+\frac{3T}{4}\right)}{\sqrt{a-I}}\right)-\operatorname{arccoth}\left(\frac{V_{\mathrm{th}}}{\sqrt{a-I}}\right)\right].$$

To compute the derivative of TTS with respect to t^{in}, we first compute several intermediate derivatives. For brevity, define $V_1:=V\left(t^{in}+\frac{T}{4}\right)=\sqrt{a-I}\,\coth A_1$ and $V_2:=V\left(t^{in}+\frac{3T}{4}\right)=\sqrt{I}\,\tan A_2$. Then

$$\begin{aligned} \frac{dA_1}{dt^{in}}&=\frac{d}{dt^{in}}\left(\operatorname{arcoth}\frac{V_r}{\sqrt{a-I}}-\sqrt{a-I}\left(t^{in}+\frac{T}{4}\right)\right)=-\sqrt{a-I},\\ \frac{dV_1}{dt^{in}}&=\frac{d}{dt^{in}}\left(\sqrt{a-I}\,\coth A_1\right)=\sqrt{a-I}\,(-\operatorname{csch}^2 A_1)\,\frac{dA_1}{dt^{in}}=(a-I)\operatorname{csch}^2 A_1\\ &=V_1^2-(a-I), \end{aligned}$$

where we used the identity $\coth^2 A_1-\operatorname{csch}^2 A_1=1$.

Since $A_2=\sqrt{I}\,\frac{T}{2}+\tan^{-1}\left(V_1/\sqrt{I}\right)$,

$$\begin{aligned} \frac{dA_2}{dt^{in}}&=\frac{1}{1+(V_1/\sqrt{I})^2}\cdot\frac{1}{\sqrt{I}}\frac{dV_1}{dt^{in}}=\frac{\sqrt{I}}{I+V_1^2}\,\frac{dV_1}{dt^{in}},\\ \frac{dV_2}{dt^{in}}&=\sqrt{I}\,\sec^2 A_2\,\frac{dA_2}{dt^{in}}=\frac{I\,\sec^2 A_2}{I+V_1^2}\,\frac{dV_1}{dt^{in}}=\frac{I+V_2^2}{I+V_1^2}\left(V_1^2-(a-I)\right), \end{aligned}$$

using the identity $\sec^2 A_2=1+\tan^2 A_2=1+V_2^2/I$.

Finally, differentiating TTS with respect to t^{in} gives

$$\begin{aligned}\frac{d}{dt^{in}}\mathrm{TTS}(t^{in}) &= 1 + \frac{1}{\sqrt{a-I}} \cdot \frac{d}{dt^{in}}\left[\operatorname{arcoth}\left(\frac{V_2}{\sqrt{a-I}}\right)\right] \\ &= 1 + \frac{1}{\sqrt{a-I}} \cdot \frac{\sqrt{a-I}}{(a-I)-V_2^2} \cdot \frac{dV_2}{dt^{in}} \\ &= 1 - \frac{\frac{dV_2}{dt^{in}}}{V_2^2-(a-I)} \\ &= 1 - \frac{I+V_2^2}{I+V_1^2}\,\frac{V_1^2-(a-I)}{V_2^2-(a-I)},\end{aligned}$$

where we used $\frac{d}{dx}\operatorname{arcoth}(x) = \frac{1}{1-x^2}$. For a fixed point t^{in}_{ss} of the map to be stable, we require $|\frac{d}{dt^{in}}\mathrm{TTS}(t^{in}_{ss})| < 1$. Recall that $\pm\sqrt{a-I}$ are the fixed points of the ode with $\tilde{E} = I - a$. Since we are assuming that the neuron fires while inhibition is on, the voltage must be blocked by the stable fixed point when inhibition is on at the start of the cycle ($V_1 < -\sqrt{a-I}$) and must have already exceeded the unstable fixed point when inhibition turns on again later in the cycle ($V_2 > \sqrt{a-I}$). Hence, the fractional term is always positive, and we have $\frac{d}{dt^{in}}\mathrm{TTS}(t^{in}) < 1$. The condition $\frac{d}{dt^{in}}\mathrm{TTS}(t^{in}) > 0$ is equivalent to

$$\frac{I+V_2^2}{I+V_1^2}\,\frac{V_1^2-(a-I)}{V_2^2-(a-I)} < 1,$$

which simplifies to

$$\frac{V_1^2-(a-I)}{I+V_1^2} < \frac{V_2^2-(a-I)}{I+V_2^2}.$$

Since the function $f(x) = \frac{x^2-(a-I)}{I+x^2}$ is monotonically increasing for $x > 0$, this condition is satisfied if and only if $V_1 < V_2$. During the inhibition-off period from $t^{in} + T/4$ to $t^{in} + 3T/4$, the neuron evolves under $V' = I + V^2$ with $I > 0$, so $V' > 0$ and the voltage is strictly increasing. Therefore, $V_1 < V_2$ is automatically satisfied, ensuring that the inhibition-off fixed point is stable.

Appendix C – Parameter sweep of steady-state phase relationships. Here we validate the analytical existence conditions for phase-locked solutions derived in Appendices A and B. For each combination of inhibition amplitude a and excitatory drive I, we numerically computed the time-to-spike function $TTS(t^{in})$ by integrating the voltage equation across a grid of initial phase values and identified stable fixed points satisfying $TTS(t^{in}_{ss}) = T$ with $0 < TTS'(t^{in}_{ss}) < 2$. The theoretically derived existence boundaries (red lines) closely match the numerically observed boundaries of phase-locking, confirming the correctness of the analytical conditions. For LIF neurons, the boundaries are $V(T; T/4) = V_{\mathrm{th}}$ (solid) and $V(T; 3T/4) = V_{\mathrm{th}}$ (dashed). For QIF neurons, the boundaries are $V(T; T/4) = V_{\mathrm{th}}$ (dashed) and $V(T; -T/4) = V_{\mathrm{th}}$ (solid).

LIF neurons (S1 Fig, panel A) exhibit phase values predominantly ranging from 90° to 270°, demonstrating their general preference for anti-phase relationships across most parameter combinations where stable solutions exist. In contrast, QIF neurons (S1 Fig, panel B) show a phase range from −90° to 130°, with most values in the middle part of this range, indicating a strong and consistent tendency toward in-phase relationships. These phase preferences are not confined to specific parameter choices but represent fundamental properties of the respective model neuron types, as demonstrated by the

consistent separation between LIF and QIF phase distributions across the full parameter space explored.

The theoretically derived existence boundaries (red lines) closely match the numerically observed phase transitions, validating the analytical conditions derived in Appendices A and B. These boundaries predict where phase-locking ceases to exist ($V(T;\, T/4) = V_{\mathrm{th}}$, solid line) and where the transition between anti-phase and in-phase regimes occurs ($V(T;\, -T/4) = V_{\mathrm{th}}$, dashed line for QIF; $V(T;\, 3T/4) = V_{\mathrm{th}}$, dashed line for LIF).

Appendix D – Supporting figures for TTS. The return map $t^{in}_{n+1} = T + t^{in}_{n} - \mathrm{TTS}(t^{in}_{n})$ provides a geometric view of the convergence to phase-locked solutions shown in Fig 7. For the LIF model (S2 Fig, panel A), the map contracts onto the stable anti-phase fixed point in the inhibition-off interval. For the QIF model (S2 Fig, panel B), it contracts onto the stable in-phase fixed point in the inhibition-on interval. These contrasting fixed-point locations directly underlie the opposite phase preferences of LIF and QIF neurons reported in the main text.

Appendix E – Single neuron dynamics under sinusoidal inhibition. The analytical results in the main text were derived under square-wave inhibition, which yields closed-form expressions for the TTS function. Here we verify that the contrasting phase preferences of LIF and QIF neurons persist when inhibition takes the more biologically realistic form of a sinusoid. As shown in S4 Fig, the qualitative structure of the TTS functions from the square-wave case is preserved: the LIF neuron retains a stable anti-phase fixed point (panels A–B) and the QIF neuron retains a stable in-phase fixed point (panels C–D). The parameter sweep in S5 Fig further confirms that these preferences hold across a wide range of excitatory drives I and inhibition amplitudes a for the LIF and QIF models and for EIF as well, closely mirroring the square-wave results in Fig 9. Together, these results confirm that the opposite phase preferences of LIF and QIF/EIF neurons are a robust consequence of their spike-generation mechanisms, not an artifact of the square-wave idealization of their inhibitory input.
