# Supplementary material for: Reconciling contradictory models of subthalamic nucleus contributions to basal ganglia beta oscillations
Source: PLoS Comput Biol. 2026 Aug 17;22(8):e1013942. doi: 10.1371/journal.pcbi.1013942 (PMC13541254; doi:10.1371/journal.pcbi.1013942)
Supplement: Supplementary file 2 [file pcbi.1013942.s008.pdf]

We thank the reviewers for their thoughtful comments and suggestions. We very much appreciate the opportunity to revise and resubmit our paper with updates to address these comments. Our detailed responses appear below.

Reviewers' comments:

Reviewer #1: This study examines how the choice of single-neuron model — specifically Leaky Integrate-and-Fire (LIF) versus Quadratic Integrate-and-Fire (QIF) — shapes emergent oscillatory dynamics in basal ganglia circuits, with a focus on the subthalamic nucleus (STN) and its role in beta band oscillations. Investigating the apparently contradictory results of two recent modeling studies (Ortone et al. 2023; Lindi et al. 2024), the authors report that LIF models yield anti-phase beta oscillations between GPe and STN, whereas QIF models produce in-phase beta oscillations. Consequently, increased STN activation reduces beta power in the LIF configuration but enhances it in the QIF configuration.

While the perspective of the study is definitely interesting, several aspects require further clarification and strengthening to render its conclusions fully compelling. Below I outline specific concerns and suggestions for revision.

Major comments:

1. In the Introduction, the authors claim that Crompe et al. showed that "STN suppression has a minimal influence on GPe power." This phrasing should be revised. Crompe et al. demonstrate that strong beta oscillations persist following STN suppression, but significant reductions in oscillatory power are reported (see Supplementary Table 2 of that study). The wording should be adjusted to accurately reflect this partial yet statistically supported effect.

Reply: We have adjusted the wording in line 24 to accurately reflect the experimental findings.

2. In the initial section of the Results, the authors state: "We initialize the model such that the pallidostriatal loop (D2→Proto→FSI→D2) generates baseline beta oscillations, while the subthalamopallidal circuit (STN/Proto) provides modulatory input." It is unclear whether in this frame the STN/Proto loop, in isolation, is capable of sustaining autonomous beta oscillations. This point should be clarified explicitly.

Reply: We have added the phrase "which does not oscillate on its own" after mentioning the subthalamopallidal circuit in line 215, which provides the requested explicit clarification.

3. Figures 3 and 4 appear to present results obtained in the absence of the Proto→STN connection. This omission should be clearly indicated, ideally via a schematic of the (sub)network configuration examined. More importantly, the analysis should be repeated—or appropriately qualified—with the Proto→STN connection included, particularly given its demonstrated relevance later in the manuscript.

Reply: In fact, the Proto→STN connection is present in both of these figures. We have revised the text about both figures in the Results section (in several places starting from line 222) as well as the figure captions to clarify this point.

4. The comparison between LIF and QIF is compelling, but the authors note that Ortone et al. (2023) actually employed an Exponential Integrate-and-Fire (EIF) model. The EIF is currently introduced only to be set aside, with only partial replication of the LIF/QIF analyses. To provide a complete and rigorous comparison, the EIF results should be integrated into Figures 6–8 and, unless they fully coincide with QIF dynamics, also into subsequent figures and analyses. This would substantially strengthen the study's conclusions regarding model-dependent effects.

The reviewer raises an excellent point about the use of the EIF model by Ortone et al. To provide the complete comparison as the reviewer suggests, we added a new subsection on the EIF model to our results section, starting on line 364. In this subsection, we introduce the EIF model and we present and discuss the new Figure 8. This new figure shows the relation of membrane potential dynamics to inhibitory input in the single neuron EIF model (Fig. 8A), analogous to the QIF result in Fig. 6A; the phase plane view of EIF voltage dynamics (Fig. 8B), analogous to the QIF result in Fig. 6B; and the EIF TTS curve (Fig. 8C), analogous to the QIF TTS in Fig. 7D. We have also added an EIF heat map as a new panel in Figure 9 (Fig. 9C), which strongly resembles that of the QIF model, and have inserted text about these EIF data in the Results section where Figure 9 is discussed (starting in line 413).

5. Lines 409–411 state: "This convergent behavior across QIF and EIF models suggests that STN neurons which are known to exhibit rapid spike initiation kinetics, would likely demonstrate in-phase coupling with Proto populations during beta oscillations." The claim that STN neurons possess "rapid spike initiation kinetics" requires supporting references. The Discussion later seems to acknowledge that such data are inconclusive or may differ between primates and rodents. This inconsistency should be resolved, and the claim either substantiated or appropriately qualified.

Reply: These lines have been removed, as they were no longer needed thanks to our updated analysis.

6. Nonlinear dynamics in the QIF model warrant further examination – Figure 13 reveals pronounced nonlinear dynamical patterns in the QIF model—particularly in the absence of heterogeneity—at firing rates of 15–20 spikes/s, which fall within the physiological range for STN neurons. This phenomenon merits deeper investigation and discussion, as it may have important implications for model selection and interpretation.

Reply: In the QIF model, as the STN firing rate increases from 0, it starts to influence Proto more strongly and, since it fires in synch with Proto as our analysis has shown, it causes the beta amplification. The reason that the beta power comes back down is that if the STN goes fast enough, it becomes out-of-phase with Proto, as we see in Fig. 13B, and hence the beta power in Proto comes back down. We have updated the text in

the Results section about Figure 13, in lines 559-575, and have rewritten the Figure 13 caption to clarify these points.

Minor comment: The manuscript would benefit from a schematic overview of the different network configurations used across figures, to aid the reader in tracking which connectivity underlies each result.

Reply: Because Reviewer 3 commented that our manuscript was already too long, and this suggestion, which would have further extended the paper, was described as a "Minor comment", we decided not to add this additional overview. We hope that this is okay and that we were able to clarify the setting for each figure through our text edits.

Reviewer #2: The manuscript studies the role of the subthalamic nucleus (STN) in the generation of pathological beta oscillations in computational models of Parkinson's disease. Motivated by conflicting results in the literature, the authors investigate how the choice of neuron model used to represent STN neurons affects network dynamics. Using rate models and spiking simulations of basal ganglia circuits, they compare leaky integrate-and-fire (LIF) and quadratic integrate-and-fire (QIF) neurons and analyze the resulting phase relationships between STN and globus pallidus prototypical neurons. They report that QIF neurons lead to in-phase coupling that amplifies beta oscillations, whereas LIF neurons produce anti-phase dynamics that suppress beta power.

I find the main idea of the manuscript interesting. In particular, the work highlights that the choice of neuron model can significantly affect the conclusions drawn about the role of the STN in beta oscillations. This is a useful observation and serves as a cautionary message regarding the impact that modeling assumptions can have on the interpretation of computational studies.

Reply: We are glad that the reviewer found our main idea to be interesting and potentially useful.

However, in my opinion this central idea is only explored at an initial stage, and the analysis could be developed further to make the conclusions more solidly grounded. Below I outline several aspects that, in my view, could strengthen the manuscript. I therefore believe that major revisions would be required before the manuscript could be considered for publication in PLOS CB.

1) In the mathematical analysis the authors consider an inhibitory signal consisting of a square wave. As I understand it, this choice allows the authors to define the TTS map analytically and derive explicit expressions for fixed points and their stability. However, it is not entirely clear to me what additional insight is gained from these analytical expressions. At the same time, the square-wave inhibition represents a strong simplification of the input received by the neurons. It would be useful to explore whether the reported effects persist under more realistic forms of inhibition, even if this requires numerical rather than analytical treatment.

Reply: We have repeated the analysis with sinusoidal inhibition, which captures the gradual onset, intensification, and offset of an inhibitory signal coming from a neuronal

population. We found that the shapes of the time-to-spike functions are analogous to those for square-wave inhibition. Moreover, the heatmaps computed with sinusoidal inhibition show that the same phase relations for the LIF (antiphase) and QIF/EIF (in-phase) model solutions those obtained in the square-wave case. We have added a new Appendix, Supplemental Section 5 (S5), referenced in lines 429-431 of the main text, where we detail these findings. This section includes two new figures, Figures 17 and 18, to accompany and illustrate these results.

2) It also seems that the problem could be analyzed from the perspective of phase response curves (PRCs). Since the manuscript studies the phase-locking of LIF and QIF neurons to a periodic inhibitory signal, it would be interesting to relate the observed differences to the shape of the corresponding PRCs. Such a connection could help clarify the mechanisms underlying the different phase relationships and place the results within the broader theoretical framework used to study synchronization in neuronal oscillators. The authors may wish to comment on this point or discuss how their findings relate to PRC-based analyses.

Reply: We are working in a strong coupling regime, which is outside of the scope of the PRC analysis. We have added a comment to this effect in the Discussion, lines 626-631, to clarify this point for all readers.

3) I find the role of the time constants in the observed phase relationships somewhat unclear. Phase-locking behavior often depends strongly on the relationship between the intrinsic periods of the oscillators when they are considered in isolation. In this context, the time constants of the spiking neuron models may play an important role in determining the phase relationship with respect to the inhibitory signal. It would therefore be helpful if the authors explored this aspect more systematically and clarified how the relevant time scales influence the resulting phase dynamics.

Reply: In the single neuron analysis, we consider non-dimensionalized dynamics. Thus, the effect of changing the membrane time constant would be reflected in changes to the inhibition period. We have added an I versus T heatmap as the new Figure 16 in the supplementary material, referenced in lines 426-429 in the Results, to illustrate the effects of these changes.

4) An important point that, in my opinion, is currently missing from the manuscript is a discussion of how real neurons compare to the simplified models considered here. Since the central conclusion of the paper is that the mathematical structure of the neuron model can qualitatively change the network behavior, it would be particularly valuable to assess which modeling framework better reflects the behavior of biological STN and pallidal neurons. For this reason, I believe the manuscript would benefit from simulations using conductance-based models that more closely follow the known biophysics of these cells. While the authors briefly mention this direction as future work in the Discussion, incorporating such analysis in the present study would considerably strengthen the conclusions and make the contribution more suitable for publication in PLOS CB.

Reply: We understand the reviewer's interest in linking our ideas to biological STN and pallidal neurons. For the purposes of this paper, however, we want to keep the focus on the two modeling frameworks already used in the literature, including in a previous *PLoS Computational Biology* study. This focus allows us to explain the discrepancy between the published results and to explain our findings in terms of the dynamic mechanisms involved. Because we performed a thorough analysis, the paper is already rather long (as pointed out by Reviewer 3). Thus, rather than adding a major extension to this manuscript, we have still left this step for future work.

5) I have some confusion regarding the definition of t_n^{in}. In line 103, t_n^{in} is defined in the interval [0,T), and the text states that it "represents the time difference between the n-th firing event and the center of the subsequent inhibition period," which suggests that it is always taken to be positive. However, later in the manuscript (lines 305–306) it is stated that t_{ss}^{in} takes values in the interval $(-T/4, 3T/4)$. Similarly, when this quantity is expressed in degrees (line 341 and Figure 8), the range becomes -90deg to 270deg. Furthermore, in Appendix S2 (line 704) it is stated that $t_{in} \in (-T/4, T/4)$, and that it is positive if the firing occurs before the midpoint of the inhibition-on interval and negative otherwise. These statements appear to use different conventions for the definition and range of t_{in}. I would appreciate it if the authors could clarify this point and ensure that the definition is consistent throughout the manuscript. This issue may also affect the computations of t_{ss} presented in the Appendix.

Reply: Thank you for your careful reading. As you pointed out, our definitions did feature some inconsistencies across settings. We have updated the text in multiple places and have thoroughly checked that the inconsistencies have been resolved. In particular, we have rewritten the text on lines 122-129 in the Methods subsection where t_n^{in} is first defined, we have made additional updates in that same subsection to maintain consistency, we have made minor related edits in lines 345-6 in the Results, and we have made minor revisions in the Appendices.

Minor:

Table 2. Regarding the inhibitory connections, should the sign of g be negative to be consistent with Equation (1)?

Reply: Thank you for catching this error. We have changed those g values in Table 2 that correspond to inhibition to be negative.

Line 105. t_{in} but t_n^{in} in the formula for $I_{inh}(t)$?

Reply: Thanks for the catch. t_{in} has been replaced by t_n^{in}.

Fig 2. The arrows in the pallidostriatal loop should go in the opposite direction. That is D2− >Proto− >FSI− >D2

Reply: Thanks for also catching this issue! We were embarrassed when we noticed this after submission and figured that a reviewer would mention it as well. The figure is now fixed.

Line 308. What does k=1 refer to in Eq (4)?

Reply: We have edited what was equation (4) and is now equation (6). The symbol k no longer appears in that equation.

Fig 8. Choice of color palette make it difficult to appreciate differences around 90deg, a boundary for fixed point ocurring at inhibition on or off.

Reply: This is now Figure 9. The color palette in this figure and in an analogous new figure, Figure 16, have been changed to emphasize the boundary between in-phase and anti-phase regimes.

Line 653. Section xx?

Reply: The appropriate section information has been provided here, which now corresponds to lines 682-3.

Line 669. I think that in the last line the exponent of e should be -T/4 instead of -T/2 at both numerator and denominator.

Reply: This expression, which now appears on line 686, has been fixed.

Line 730. Why without loss of generality you can choose Vth=1? Apparently, it seems that depending on how V_{th} is chosen it may affect whether condition (17) or (18) are satisfied or not.

Reply: What we meant here is that since we do not have any constraints on the parameter values that we consider in this example, we might as well simplify things and fix one parameter to 1. To phrase it differently, any conclusions we draw for $V_{th} = 1$ could be rescaled to similar conclusions for any other value of V_{th}. We have changed the wording slightly in line 713-4 of our manuscript to clarify our meaning.

Line 732. inequalty Line 753. equivlent

Reply: Thanks for your careful reading. We have fixed these typos.

Reviewer #3: Summary: This is a very nice paper that resolves an apparent mismatch between two recent papers about the origin of beta band oscillations in the basal ganglia. There are multiple loops in the basal ganglia (Zang et al. eLIFE 2024) that can generate oscillations on their own. However, an interesting question is how two or more oscillators interact. A recent paper showed that when STN-PROTO loop interacts with the D2-FS-Proto loop power beta power is decreased while another showed the opposite for the same system. The answer is of course in the two papers. One used the exponential integrate and fire model while the other used the leaky integrate and fire model. However, it remained to be shown whether the difference was due to neuron model or some other details of network connectivity. Here authors pretty neatly demonstrate (although there are some concerns) that the differences can be attributed to the choice of neuron model. This is a very nice demonstration of how single neuron property could manifest at network level. The paper is very well written and illustrated. The proofs seem correct (although I did not attempt to derive myself). But there are some concerns that need to be addressed. Mainly I am wondering if the amplification due to QIF and attenuation due to the LIF

neurons model could not be explained by the small mismatch in the oscillation frequencies.

Reply: Thank you very much for your positive feedback and for sharing your suggestions.

Questions: - What was the oscillation frequency of STN-Proto (with lif and lif) and D2-FS-Proto loops individually? When the delay is introduced STN-Proto loop frequency will be affected. In fact it does seem that in with-delay the oscillation frequency is somewhat reduced. The change in the power could be understood in terms of the mismatch between the frequency of two oscillators?

Reply: In fact, our STN-Proto loop does not oscillate on its own. Thus, there is no question of a mismatch in the oscillation frequency between the two loops in the regime that we consider. We have added some text to the second and third paragraphs of the Results section to clarify this point.

- Figure 12 very nicely illustrates the effect. I think here we can see the issue with the frequency. My suspicion is that the oscillations frequency in the LIF and QIF are slightly different and that's what is driving the effect. So it would be good to clearly show that the STN-PROTO network with QIF and LIF neurons has the same frequency as the proto-f2-d2 loop. And that the effect cannot be explained by the frequency mismatch.

Reply: If we understand the reviewer correctly, then the absence of oscillations in the isolated STN-Proto loop takes care of this comment as well. We also point out to the reviewer that in what had been Figure 12 and is now Figure 13, we vary the firing frequency of the STN itself, in both modeling frameworks. We see that even when the STN has the same frequency, the LIF and QIF results can differ substantially.

- How does the oscillation frequency change when I_{proto} and g-proto-stn are varied.

Reply: We believe that this is intended as an extension of the two previous questions. Nonetheless, we went ahead and computed the Proto oscillation frequency under these parameter variations. We found that the frequency remained in the beta band, between 17 and 22 Hz. Given that the Proto-FSI-D2 loop produces oscillations that are then transferred to the STN, the relative invariance of Proto oscillation frequency to variations in these parameters makes sense. We now show a heat map of Proto oscillation frequency in Fig. 4B, alongside the heat map of Proto oscillation amplitude in Fig. 4A, and have added a few lines of text (lines 256-9) to refer to these results.

- I am not sure how the network is setup so that it gives maximal oscillation power for zero input (see Figure 4A). STN photo connection is set to zero and D2 projections are also inhibitory. Please clarify.

Reply: Our overall model parameters are set such that the Proto component oscillates with $I_{Proto} = 0$. This level of drive can be thought of as a baseline for the Proto unit.

- Single neuron dynamics: Most of the parameters for the results shown in figure 6 are missing. I am not sure how general the results are. It must depend on the time constant,

amplitude of the excitatory and inhibitory inputs (I_1 or I_2 and I_{inh}) and the frequency of inhibitory pulse. The proof also exclude the time constant of the neurons and ignores if the neuron may have an intrinsic resonance frequency.

Reply: We have edited the *Single neuron models* part of the Methods section, starting on line 94, to provide the previously omitted parameter values. As noted in our response to comment 3 by Reviewer #2, in the single neuron analysis, we consider non-dimensionalized dynamics. Thus, the effect of changing the membrane time constant is reflected in changes to the inhibition period, T. We have added an I versus T heatmap as the new Figure 16 in the supplementary material, referenced in lines 426-9 in the Results, to illustrate the effects of these changes. More generally, Figures 4, 9, 10C, 13, 14, 16, and 18 all relate to the robustness of our key results with respect to parameter variations.

As for the proofs in the first two appendices, what is of central importance are the fundamental dynamical mechanisms associated with the LIF and QIF models. We have not omitted any features of these models from our analysis. Since we do derive formulas in these calculations in terms of model parameter values, we have added text at the start of each of these appendices to note that we have rescaled time to units of the membrane time constant.

- In Figure 6A spike phase is fluctuating but in 7C it seems to reach a steady state (there may be beats forming due to slight mismatch between inhibitory frequency and neuron spiking frequency). These two examples suggest that the TTS convergence is dependent on model parameters. It would be nice to see a panel similar to 7E for QIF neuron.

Reply: We have added Figure 7F to provide the requested illustration.

- The paper is quite long but a figure that may be added is the return map of the TTS for the three neuron models used in the paper. Similarly a figure showing the dependence of the stable solution in an and I would be helpful to a reader to get an idea of the relative inputs and inhibition needed.

Reply: The new Figure 15 in the supplemental material shows the return maps for the LIF and QIF models. Since these are determined by the TTS function, and we have added Figure 8C to show that the TTS for EIF is similar to that for QIF, we felt it would be repetitive to also show the similar return map for EIF. We apologize but we aren't sure how to parse the final part of the reviewer's comment, about the stable solution. Hopefully the information of interest is now provided in the supplement in any case (e.g., Figure 14 shows where oscillations occur, and what their stable phase relations are, as inhibition amplitude a and drive I to Proto are varied).

- It would be good to provide a short text to explain how TTS may relate to PRC and whether the different results of the two papers could not be explained using the shape of the phase response curve?

Reply: As noted in our response to Reviewer #2, comment 2, we are working in a strong coupling regime, so PRC analysis does not apply. We have added a comment to the Discussion, lines 626-31, to clarify this point for readers.

- While I appreciate the pedagogic nature of the manuscript, there is not much new information up to the first 9 figures. All these results could be presented in a more compact form. The point is clear that the QIF neuron has a faster rise in the membrane potential closer to the threshold and therefore QIF neurons can also fire during the inhibitory phase of the proto input, while LIF may not.

Reply: Looking at the comments of the three reviewers as a whole, including requests for additional figure panels for some of these early figures, questions about robustness and effects of parameter variations, and other comments asking for more, not less, information about some of the points made there, we made the judgment call that it's better to keep these figures in the main document. While the paper is long, readers who are already familiar with these ideas or are less interested in the details can easily skip parts of the exposition.
