# Supplementary material for: Reconciling contradictory models of subthalamic nucleus contributions to basal ganglia beta oscillations
Source: PLoS Comput Biol. 2026 Aug 17;22(8):e1013942. doi: 10.1371/journal.pcbi.1013942 (PMC13541254; doi:10.1371/journal.pcbi.1013942)
Supplement: Supplementary file 3 [file pcbi.1013942.s009.pdf]

We are pleased that 2 reviewers feel our paper is ready for publication and are glad to address the remaining comments from Reviewer #3. As noted below, we have made some minor revisions to our manuscript related to these comments. These revisions appear in lines 70-75 of the manuscript.

Reviewer #3:

-My first concern was whether there is a difference in the oscillation frequency of the two loops. Authors now note that STN-PROTO loop does not oscillate. This is not a correct assumption. But I can imagine that the network does not oscillate for the chosen inputs but the network must have an intrinsic oscillation frequency. So the question of mismatch of the oscillation frequencies still stands and demands an explanation.

There are two parts to the answer to this question. First, there is the question of whether the STN-Proto loop oscillates. In fact, because the STN (excitatory) population lacks recurrent connections, the firing rate model for the STN-Proto loop simply does not oscillate. We have updated the text in the revised document to point out this absence of recurrent excitation. Now, it is true that a network of spiking E and I neurons may generate oscillations with appropriate parameter choices, but this leads to the second, and most important, part of our answer: Our single neuron analysis, in which we drive different STN model neurons with an extraneous, periodic input, shows that different STN models phase lock to their inputs with different phase relationships. Thus, the mechanisms for the distinct forms of phase-locking and their associated impact on beta oscillations are present in the single neuron dynamics, rather than emerging from a latent intrinsic oscillation frequency of the STN-Proto loop.

- In response to my concerns about maximum oscillation power at zero input authors replied that "Our overall model parameters are set such that the Proto component oscillates
with IP roto = 0. This level of drive can be thought of as a baseline for the Proto unit." This makes me wonder about the whole operating range of the model. How could you claim that baseline drive to Proto neurons is zero?

We apologize for causing confusion. We did not mean to imply that there is no drive to biological Proto neurons. We were referring to baseline parameter choices in our abstract neuronal models. To be more clear: In equation (1), c_p for each neuronal population sets a firing rate in the absence of inputs ($b_p=0$), b_p sets a sensitivity to inputs, a_p is a gain scaling parameter, and I_p serves as an extra tuning

parameter. We used these parameters to tune the abstract model (1) to match certain properties of the biological neurons, such as the baseline and average firing rates. In our manuscript, we have revised the wording to clarify that (1) is an abstract model and that its parameters do not have a detailed biological interpretation but rather are used to achieve reasonable firing rates for each population.
