# Supplementary material for: Reconciling contradictory models of subthalamic nucleus contributions to basal ganglia beta oscillations
Source: PLoS Comput Biol. 2026 Aug 17;22(8):e1013942. doi: 10.1371/journal.pcbi.1013942 (PMC13541254; doi:10.1371/journal.pcbi.1013942)
Supplement: Peer review history

# Peer review: Decision Letter 0

**DOI:** 10.1371/journal.pcbi.1013942.r001

**Authors:** Lyle Graham, Kim Blackwell

10 Mar 2026

Reconciling contradictory models of subthalamic nucleus contributions to basal ganglia beta oscillations

PLOS Computational Biology

Dear Dr. Rubin,

Thank you for submitting your manuscript to PLOS Computational Biology. After careful consideration, we feel that it has merit but does not fully meet PLOS Computational Biology's publication criteria as it currently stands. Therefore, we invite you to submit a revised version of the manuscript that addresses the points raised during the review process.

Please submit your revised manuscript by May 10 2026 11:59PM. If you will need more time than this to complete your revisions, please reply to this message or contact the journal office at ploscompbiol@plos.org. When you're ready to submit your revision, log on to https://www.editorialmanager.com/pcompbiol/ and select the 'Submissions Needing Revision' folder to locate your manuscript file.

Please include the following items when submitting your revised manuscript:

* A letter that responds to each point raised by the editor and reviewer(s). You should upload this letter as a separate file labeled 'Response to Reviewers'. This file does not need to include responses to formatting updates and technical items listed in the 'Journal Requirements' section below.

* A marked-up copy of your manuscript that highlights changes made to the original version. You should upload this as a separate file labeled 'Revised Manuscript with Track Changes'.

* An unmarked version of your revised paper without tracked changes. You should upload this as a separate file labeled 'Manuscript'.

If you would like to make changes to your financial disclosure, competing interests statement, or data availability statement, please make these updates within the submission form at the time of resubmission. Guidelines for resubmitting your figure files are available below the reviewer comments at the end of this letter

We look forward to receiving your revised manuscript.

Kind regards,

Kim T. Blackwell, V.M.D., Ph.D.

Academic Editor

PLOS Computational Biology

Lyle Graham

Section Editor

PLOS Computational Biology

**Journal Requirements:**

1) Please ensure that the CRediT author contributions listed for every co-author are completed accurately and in full.

At this stage, the following Authors/Authors require contributions: Ka Nap Tse, G. Bard Ermentrout, and Jonathan Rubin. Please ensure that the full contributions of each author are acknowledged in the "Add/Edit/Remove Authors" section of our submission form.

The list of CRediT author contributions may be found here: https://journals.plos.org/ploscompbiol/s/authorship#loc-author-contributions

2) We ask that a manuscript source file is provided at Revision. Please upload your manuscript file as a .doc, .docx, .rtf or .tex. If you are providing a .tex file, please upload it under the item type u2018LaTeX Source Fileu2019 and leave your .pdf version as the item type u2018Manuscriptu2019.

3) Please upload all main figures as separate Figure files in .tif or .eps format. For more information about how to convert and format your figure files please see our guidelines:

https://journals.plos.org/ploscompbiol/s/figures

4) We notice that your supplementary information is included in the manuscript file. Please remove them and upload them with the file type 'Supporting Information'. Please ensure that each Supporting Information file has a legend listed in the manuscript after the references list.

Note: If the reviewer comments include a recommendation to cite specific previously published works, please review and evaluate these publications to determine whether they are relevant and should be cited. There is no requirement to cite these works unless the editor has indicated otherwise.

**Reviewers' comments:**

Reviewer's Responses to Questions

Reviewer #1: This study examines how the choice of single-neuron model — specifically Leaky Integrate-and-Fire (LIF) versus Quadratic Integrate-and-Fire (QIF) — shapes emergent oscillatory dynamics in basal ganglia circuits, with a focus on the subthalamic nucleus (STN) and its role in beta band oscillations. Investigating the apparently contradictory results of two recent modeling studies (Ortone et al. 2023; Lindi et al. 2024), the authors report that LIF models yield anti-phase beta oscillations between GPe and STN, whereas QIF models produce in-phase beta oscillations. Consequently, increased STN activation reduces beta power in the LIF configuration but enhances it in the QIF configuration.

While the perspective of the study is definitely interesting, several aspects require further clarification and strengthening to render its conclusions fully compelling. Below I outline specific concerns and suggestions for revision.

Major comments:

1. In the Introduction, the authors claim that Crompe et al. showed that “STN suppression has a minimal influence on GPe power.” This phrasing should be revised. Crompe et al. demonstrate that strong beta oscillations persist following STN suppression, but significant reductions in oscillatory power are reported (see Supplementary Table 2 of that study). The wording should be adjusted to accurately reflect this partial yet statistically supported effect.

2. In the initial section of the Results, the authors state:

“We initialize the model such that the pallidostriatal loop (D2→Proto→FSI→D2) generates baseline beta oscillations, while the subthalamopallidal circuit (STN↔Proto) provides modulatory input.”

It is unclear whether in this frame the STN↔Proto loop, in isolation, is capable of sustaining autonomous beta oscillations. This point should be clarified explicitly.

3. Figures 3 and 4 appear to present results obtained in the absence of the Proto→STN connection. This omission should be clearly indicated, ideally via a schematic of the (sub)network configuration examined. More importantly, the analysis should be repeated—or appropriately qualified—with the Proto→STN connection included, particularly given its demonstrated relevance later in the manuscript.

4. The comparison between LIF and QIF is compelling, but the authors note that Ortone et al. (2023) actually employed an Exponential Integrate-and-Fire (EIF) model. The EIF is currently introduced only to be set aside, with only partial replication of the LIF/QIF analyses. To provide a complete and rigorous comparison, the EIF results should be integrated into Figures 6–8 and, unless they fully coincide with QIF dynamics, also into subsequent figures and analyses. This would substantially strengthen the study’s conclusions regarding model-dependent effects.

5. Lines 409–411 state:

“This convergent behavior across QIF and EIF models suggests that STN neurons which are known to exhibit rapid spike initiation kinetics, would likely demonstrate in-phase coupling with Proto populations during beta oscillations.”

The claim that STN neurons possess “rapid spike initiation kinetics” requires supporting references. The Discussion later seems to acknowledge that such data are inconclusive or may differ between primates and rodents. This inconsistency should be resolved, and the claim either substantiated or appropriately qualified.

6. Nonlinear dynamics in the QIF model warrant further examination – Figure 13 reveals pronounced nonlinear dynamical patterns in the QIF model—particularly in the absence of heterogeneity—at firing rates of 15–20 spikes/s, which fall within the physiological range for STN neurons. This phenomenon merits deeper investigation and discussion, as it may have important implications for model selection and interpretation.

Minor comment:

• The manuscript would benefit from a schematic overview of the different network configurations used across figures, to aid the reader in tracking which connectivity underlies each result.

Reviewer #2: The manuscript studies the role of the subthalamic nucleus (STN) in the generation of pathological beta oscillations in computational models of Parkinson’s disease. Motivated by conflicting results in the literature, the authors investigate how the choice of neuron model used to represent STN neurons affects network dynamics. Using rate models and spiking simulations of basal ganglia circuits, they compare leaky integrate-and-fire (LIF) and quadratic integrate-and-fire (QIF) neurons and analyze the resulting phase relationships between STN and globus pallidus prototypical neurons. They report that QIF neurons lead to in-phase coupling that amplifies beta oscillations, whereas LIF neurons produce anti-phase dynamics that suppress beta power.

I find the main idea of the manuscript interesting. In particular, the work highlights that the choice of neuron model can significantly affect the conclusions drawn about the role of the STN in beta oscillations. This is a useful observation and serves as a cautionary message regarding the impact that modeling assumptions can have on the interpretation of computational studies.

However, in my opinion this central idea is only explored at an initial stage, and the analysis could be developed further to make the conclusions more solidly grounded. Below I outline several aspects that, in my view, could strengthen the manuscript. I therefore believe that major revisions would be required before the manuscript could be considered for publication in PLOS CB.

1) In the mathematical analysis the authors consider an inhibitory signal consisting of a square wave. As I understand it, this choice allows the authors to define the TTS map analytically and derive explicit expressions for fixed points and their stability. However, it is not entirely clear to me what additional insight is gained from these analytical expressions. At the same time, the square-wave inhibition represents a strong simplification of the input received by the neurons. It would be useful to explore whether the reported effects persist under more realistic forms of inhibition, even if this requires numerical rather than analytical treatment.

2) It also seems that the problem could be analyzed from the perspective of phase response curves (PRCs). Since the manuscript studies the phase-locking of LIF and QIF neurons to a periodic inhibitory signal, it would be interesting to relate the observed differences to the shape of the corresponding PRCs. Such a connection could help clarify the mechanisms underlying the different phase relationships and place the results within the broader theoretical framework used to study synchronization in neuronal oscillators. The authors may wish to comment on this point or discuss how their findings relate to PRC-based analyses.

3) I find the role of the time constants in the observed phase relationships somewhat unclear. Phase-locking behavior often depends strongly on the relationship between the intrinsic periods of the oscillators when they are considered in isolation. In this context, the time constants of the spiking neuron models may play an important role in determining the phase relationship with respect to the inhibitory signal. It would therefore be helpful if the authors explored this aspect more systematically and clarified how the relevant time scales influence the resulting phase dynamics.

4) An important point that, in my opinion, is currently missing from the manuscript is a discussion of how real neurons compare to the simplified models considered here. Since the central conclusion of the paper is that the mathematical structure of the neuron model can qualitatively change the network behavior, it would be particularly valuable to assess which modeling framework better reflects the behavior of biological STN and pallidal neurons. For this reason, I believe the manuscript would benefit from simulations using conductance-based models that more closely follow the known biophysics of these cells. While the authors briefly mention this direction as future work in the Discussion, incorporating such analysis in the present study would considerably strengthen the conclusions and make the contribution more suitable for publication in PLOS CB.

5) I have some confusion regarding the definition of $t_n^in$. In line 103, $t_n^in$ is defined in the interval [0,T), and the text states that it “represents the time difference between the n-th firing event and the center of the subsequent inhibition period,” which suggests that it is always taken to be positive. However, later in the manuscript (lines 305–306) it is stated that $t_ss^in$ takes values in the interval (−T/4,3T/4). Similarly, when this quantity is expressed in degrees (line 341 and Figure 8), the range becomes −90deg to 270deg. Furthermore, in Appendix S2 (line 704) it is stated that

$t_in \in (−T/4,T/4)$, and that it is positive if the firing occurs before the midpoint of the inhibition-on interval and negative otherwise. These statements appear to use different conventions for the definition and range of $t_in$. I would appreciate it if the authors could clarify this point and ensure that the definition is consistent throughout the manuscript. This issue may also affect the computations of $t_ss$ presented in the Appendix.

Minor:

Table 2. Regarding the inhibitory connections, should the sign of g be negative to be consistent with Equation (1)?

Line 105. t_{in} but t_{n}^in in the formula for I_inh(t)?

Fig 2. The arrows in the pallidostriatal loop should go in the opposite direction. That is D2->Proto->FSI->D2

Line 308. What does k=1 refer to in Eq (4)?

Fig 8. Choice of color palette make it difficult to appreciate differences around 90deg, a boundary for fixed point ocurring at inhibition on or off.

Line 653. Section xx?

Line 669. I think that in the last line the exponent of e should be -T/4 instead of -T/2 at both numerator and denominator.

Line 730. Why without loss of generality you can choose Vth=1? Apparently, it seems that depending on how V_th is chosen it may affect whether condition (17) or (18) are satisfied or not.

Line 732. inequalty

Line 753. equivlent

Reviewer #3: Summary:

This is a very nice paper that resolves an apparent mismatch between two recent papers about the origin of beta band oscillations in the basal ganglia. There are multiple loops in the basal ganglia (Zang et al. eLIFE 2024) that can generate oscillations on their own. However, an interesting question is how two or more oscillators interact. A recent paper showed that when STN-PROTO loop interacts with the D2-FS-Proto loop power beta power is decreased while another showed the opposite for the same system. The answer is of course in the two papers. One used the exponential integrate and fire model while the other used the leaky integrate and fire model. However, it remained to be shown whether the difference was due to neuron model or some other details of network connectivity.

Here authors pretty neatly demonstrate (although there are some concerns) that the differences can be attributed to the choice of neuron model. This is a very nice demonstration of how single neuron property could manifest at network level. The paper is very well written and illustrated. The proofs seem correct (although I did not attempt to derive myself). But there are some concerns that need to be addressed. Mainly I am wondering if the amplification due to QIF and attenuation due to the LIF neurons model could not be explained by the small mismatch in the oscillation frequencies.

Questions:

- What was the oscillation frequency of STN-Proto (with lif and lif) and D2-FS-Proto loops individually? When the delay is introduced STN-Proto loop frequency will be affected. In fact it does seem that in with-delay the oscillation frequency is somewhat reduced. The change in the power could be understood in terms of the mismatch between the frequency of two oscillators?

- Figure 12 very nicely illustrates the effect. I think here we can see the issue with the frequency. My suspicion is that the oscillations frequency in the LIF and QIF are slightly different and that's what is driving the effect. So it would be good to clearly show that the STN-PROTO network with QIF and LIF neurons has the same frequency as the proto-f2-d2 loop. And that the effect cannot be explained by the frequency mismatch.

- How does the oscillation frequency change when I_proto and g_proto_stn are varied.

- I am not sure how the network is setup so that it gives maximal oscillation power for zero input (see Figure 4A). STN photo connection is set to zero and D2 projections are also inhibitory. Please clarify.

- Single neuron dynamics: Most of the parameters for the results shown in figure 6 are missing. I am not sure how general the results are. It must depend on the time constant, amplitude of the excitatory and inhibitory inputs (I_1 or I_2 and I_inh) and the frequency of inhibitory pulse. The proof also exclude the time constant of the neurons and ignores if the neuron may have an intrinsic resonance frequency.

- In Figure 6A spike phase is fluctuating but in 7C it seems to reach a steady state (there may be beats forming due to slight mismatch between inhibitory frequency and neuron spiking frequency). These two examples suggest that the TTS convergence is dependent on model parameters. It would be nice to see a panel similar to 7E for QIF neuron.

- The paper is quite long but a figure that may be added is the return map of the TTS for the three neuron models used in the paper. Similarly a figure showing the dependence of the stable solution in an and I would be helpful to a reader to get an idea of the relative inputs and inhibition needed.

- It would be good to provide a short text to explain how TTS may relate to PRC and whether the different results of the two papers could not be explained using the shape of the phase response curve?

- While I appreciate the pedagogic nature of the manuscript, there is not much new information up to the first 9 figures. All these results could be presented in a more compact form. The point is clear that the QIF neuron has a faster rise in the membrane potential closer to the threshold and therefore QIF neurons can also fire during the inhibitory phase of the proto input, while LIF may not.

**********

**Have the authors made all data and (if applicable) computational code underlying the findings in their manuscript fully available?**

The PLOS Data policy requires authors to make all data and code underlying the findings described in their manuscript fully available without restriction, with rare exception (please refer to the Data Availability Statement in the manuscript PDF file). The data and code should be provided as part of the manuscript or its supporting information, or deposited to a public repository. For example, in addition to summary statistics, the data points behind means, medians and variance measures should be available. If there are restrictions on publicly sharing data or code —e.g. participant privacy or use of data from a third party—those must be specified.

Reviewer #1: Yes

Reviewer #2: Yes

Reviewer #3: Yes

**********

PLOS authors have the option to publish the peer review history of their article (what does this mean?). If published, this will include your full peer review and any attached files.

If you choose “no”, your identity will remain anonymous but your review may still be made public.

**Do you want your identity to be public for this peer review?**  For information about this choice, including consent withdrawal, please see our Privacy Policy.

Reviewer #1: No

Reviewer #2: No

Reviewer #3: No

[NOTE: If reviewer comments were submitted as an attachment file, they will be attached to this email and accessible via the submission site. Please log into your account, locate the manuscript record, and check for the action link "View Attachments". If this link does not appear, there are no attachment files.]

**Figure resubmission:**

After uploading your figures to PLOS’s NAAS tool - https://ngplosjournals.pagemajik.ai/artanalysis, NAAS will process the files provided and display the results in the "Uploaded Files" section of the page as the processing is complete. If the uploaded figures meet our requirements (or NAAS is able to fix the files to meet our requirements), the figure will be marked as "fixed" above. If NAAS is unable to fix the files, a red "failed" label will appear above. When NAAS has confirmed that the figure files meet our requirements, please download the file via the download option, and include these NAAS processed figure files when submitting your revised manuscript.

**Reproducibility:**

To enhance the reproducibility of your results, we recommend that authors of applicable studies deposit laboratory protocols in protocols.io, where a protocol can be assigned its own identifier (DOI) such that it can be cited independently in the future. Additionally, PLOS ONE offers an option to publish peer-reviewed clinical study protocols. Read more information on sharing protocols at https://plos.org/protocols?utm_medium=editorial-email&utm_source=authorletters&utm_campaign=protocols

# Author comment: Author response to Decision Letter 1

**DOI:** 10.1371/journal.pcbi.1013942.r002

27 May 2026

Attachment Submitted filename: Tse_response_reviewers.pdf

# Peer review: Decision Letter 1

**DOI:** 10.1371/journal.pcbi.1013942.r003

**Authors:** Lyle Graham, Kim Blackwell

17 Jun 2026

PCOMPBIOL-D-26-00163R1

Reconciling contradictory models of subthalamic nucleus contributions to basal ganglia beta oscillations

PLOS Computational Biology

Dear Dr. Rubin,

Thank you for submitting your manuscript to PLOS Computational Biology. After careful consideration, we feel that it has merit but does not fully meet PLOS Computational Biology's publication criteria as it currently stands. Therefore, we invite you to submit a revised version of the manuscript that addresses just a few remaining points raised by one of the reviewers.

Please submit your revised manuscript by Aug 17 2026 11:59PM. If you will need more time than this to complete your revisions, please reply to this message or contact the journal office at ploscompbiol@plos.org. When you're ready to submit your revision, log on to https://www.editorialmanager.com/pcompbiol/ and select the 'Submissions Needing Revision' folder to locate your manuscript file.

Please include the following items when submitting your revised manuscript:

* A letter that responds to each point raised by the editor and reviewer(s). You should upload this letter as a separate file labeled 'Response to Reviewers'. This file does not need to include responses to formatting updates and technical items listed in the 'Journal Requirements' section below.

* A marked-up copy of your manuscript that highlights changes made to the original version. You should upload this as a separate file labeled 'Revised Manuscript with Track Changes'.

* An unmarked version of your revised paper without tracked changes. You should upload this as a separate file labeled 'Manuscript'.

If you would like to make changes to your financial disclosure, competing interests statement, or data availability statement, please make these updates within the submission form at the time of resubmission. Guidelines for resubmitting your figure files are available below the reviewer comments at the end of this letter.

As the corresponding author, your ORCID iD is verified in the submission system and will appear in the published article. PLOS supports the use of ORCID, and we encourage all coauthors to register for an ORCID iD and use it as well. Please encourage your coauthors to verify their ORCID iD within the submission system before final acceptance, as unverified ORCID iDs will not appear in the published article. Only the individual author can complete the verification step; PLOS staff cannot verify ORCID iDs on behalf of authors.

We look forward to receiving your revised manuscript.

Kind regards,

Kim T. Blackwell, V.M.D., Ph.D.

Academic Editor

PLOS Computational Biology

Lyle Graham

Section Editor

PLOS Computational Biology

**Journal Requirements:**

If the reviewer comments include a recommendation to cite specific previously published works, please review and evaluate these publications to determine whether they are relevant and should be cited. There is no requirement to cite these works unless the editor has indicated otherwise.

1) The resolution of Figure Fig 1-13 is very low and somewhat difficult to read. It is important that our Editors and Peer Reviewers are able to read all parts of a submission. Please replace this figure with a higher resolution copy.

2) We have noticed that you have uploaded Supporting Information files, but you have not included a list of legends. Please add a full list of legends for your Supporting Information files after the references list.

3) Please provide a complete Data Availability Statement in the submission form, ensuring you include all necessary access information or a reason for why you are unable to make your data freely accessible. If your research concerns only data provided within your submission, please write "All data are in the manuscript and/or supporting information files" as your Data Availability Statement

4)We have amended your Competing Interest statement to comply with journal style. We kindly ask that you double check the statement and let us know if anything is incorrect.

**Reviewers' comments:**

Reviewer's Responses to Questions

**Comments to the Authors:**

**Please note here if the review is uploaded as an attachment.**

Reviewer #1: All comments have been properly addressed

Reviewer #2: The authors have adequately addressed the reviewers' comments and revised the manuscript accordingly. The revisions have improved the clarity of the paper, and I have no further substantive concerns. I believe the paper is now suitable for publication.

Reviewer #3: Thank you for revising the manuscript. But I think two of my key concerns still stand and require explanation.

- My first concern was whether there is a difference in the oscillation frequency of the two loops. Authors now note that STN-PROTO loop does not oscillate. This is not a correct assumption. But I can imagine that the network does not oscillate for the chosen inputs but the network must have an intrinsic oscillation frequency. So the question of mismatch of the oscillation frequencies still stands and demands an explanation.

- In response to my concerns about maximum oscillation power at zero input authors replied that "Our overall model parameters are set such that the Proto component oscillates

with IP roto = 0. This level of drive can be thought of as a baseline for the Proto unit."

This makes me wonder about the whole operating range of the model. How could you claim that baseline drive to Proto neurons is zero?

**********

**Have the authors made all data and (if applicable) computational code underlying the findings in their manuscript fully available?**

The PLOS Data policy requires authors to make all data and code underlying the findings described in their manuscript fully available without restriction, with rare exception (please refer to the Data Availability Statement in the manuscript PDF file). The data and code should be provided as part of the manuscript or its supporting information, or deposited to a public repository. For example, in addition to summary statistics, the data points behind means, medians and variance measures should be available. If there are restrictions on publicly sharing data or code —e.g. participant privacy or use of data from a third party—those must be specified.

Reviewer #1: Yes

Reviewer #2: None

Reviewer #3: Yes

**********

PLOS authors have the option to publish the peer review history of their article (what does this mean?). If published, this will include your full peer review and any attached files.

If you choose “no”, your identity will remain anonymous but your review may still be made public.

**Do you want your identity to be public for this peer review?**  For information about this choice, including consent withdrawal, please see our Privacy Policy.

Reviewer #1: No

Reviewer #2: No

Reviewer #3: No

[NOTE: If reviewer comments were submitted as an attachment file, they will be attached to this email and accessible via the submission site. Please log into your account, locate the manuscript record, and check for the action link "View Attachments". If this link does not appear, there are no attachment files.]

**Figure resubmission:**

**Reproducibility:**

To enhance the reproducibility of your results, we recommend that authors of applicable studies deposit laboratory protocols in protocols.io, where a protocol can be assigned its own identifier (DOI) such that it can be cited independently in the future. Additionally, PLOS ONE offers an option to publish peer-reviewed clinical study protocols. Read more information on sharing protocols at https://plos.org/protocols?utm_medium=editorial-email&utm_source=authorletters&utm_campaign=protocols

# Author comment: Author response to Decision Letter 2

**DOI:** 10.1371/journal.pcbi.1013942.r004

1 Jul 2026

Attachment Submitted filename: ResponsesToReviewer_round2.pdf

# Editor report: Decision Letter 2

**DOI:** 10.1371/journal.pcbi.1013942.r005

**Authors:** Lyle Graham, Kim Blackwell

2 Jul 2026

Dear Dr. Rubin,

We are pleased to inform you that your manuscript 'Reconciling contradictory models of subthalamic nucleus contributions to basal ganglia beta oscillations' has been provisionally accepted for publication in PLOS Computational Biology.

Before your manuscript can be formally accepted you will need to complete some formatting changes, which you will receive in a follow up email. A member of our team will be in touch with a set of requests.

Please note that your manuscript will not be scheduled for publication until you have made the required changes, so a swift response is appreciated.

IMPORTANT: The editorial review process is now complete. PLOS will only permit corrections to spelling, formatting or significant scientific errors from this point onwards. Requests for major changes, or any which affect the scientific understanding of your work, will cause delays to the publication date of your manuscript.

Should you, your institution's press office or the journal office choose to press release your paper, you will automatically be opted out of early publication. We ask that you notify us now if you or your institution is planning to press release the article. All press must be co-ordinated with PLOS.

Thank you again for supporting Open Access publishing; we are looking forward to publishing your work in PLOS Computational Biology.

Best regards,

Kim T. Blackwell, V.M.D., Ph.D.

Academic Editor

PLOS Computational Biology

Lyle Graham

Section Editor

PLOS Computational Biology

***********************************************************

# Editor report: Acceptance letter

**DOI:** 10.1371/journal.pcbi.1013942.r006

**Authors:** Lyle Graham, Kim Blackwell

PCOMPBIOL-D-26-00163R2

Reconciling contradictory models of subthalamic nucleus contributions to basal ganglia beta oscillations

Dear Dr Rubin,

I am pleased to inform you that your manuscript has been formally accepted for publication in PLOS Computational Biology. Your manuscript is now with our production department and you will be notified of the publication date in due course.

The corresponding author will soon be receiving a typeset proof for review, to ensure errors have not been introduced during production. Please review the PDF proof of your manuscript carefully, as this is the last chance to correct any errors. Please note that major changes, or those which affect the scientific understanding of the work, will likely cause delays to the publication date of your manuscript.

Soon after your final files are uploaded, unless you have opted out, the early version of your manuscript will be published online. The date of the early version will be your article's publication date. The final article will be published to the same URL, and all versions of the paper will be accessible to readers.

For Research, Software, and Methods articles, you will receive an invoice from PLOS for your publication fee after your manuscript has reached the completed accept phase. If you receive an email requesting payment before acceptance or for any other service, this may be a phishing scheme. Learn how to identify phishing emails and protect your accounts at https://explore.plos.org/phishing.

Thank you again for supporting PLOS Computational Biology and open-access publishing. We are looking forward to publishing your work!

With kind regards,

Sharmila Kamatchi

PLOS Computational Biology | Carlyle House, Carlyle Road, Cambridge CB4 3DN | United Kingdom ploscompbiol@plos.org | Phone +44 (0) 1223-442824 | ploscompbiol.org | @PLOSCompBiol
